# Vitamin B_12_ protects necrosis of acinar cells in pancreatic tissues with acute pancreatitis

**DOI:** 10.1002/mco2.686

**Published:** 2024-10-15

**Authors:** Yulin Chen, Xue Li, Ran Lu, Yinchun Lv, Yongzi Wu, Junman Ye, Jin Zhao, Li Li, Qiaorong Huang, Wentong Meng, Feiwu Long, Wei Huang, Qing Xia, Jianbo Yu, Chuanwen Fan, Xianming Mo

**Affiliations:** ^1^ West China Center of Excellence for Pancreatitis Institute of Integrated Traditional Chinese and Western Medicine Laboratory of Stem Cell Biology State Key Laboratory of Biotherapy West China Hospital, Sichuan University Chengdu China; ^2^ Department of Occupational and Environmental Health West China School of Public Health and West China Fourth Hospital Sichuan University Chengdu China; ^3^ West China‐PUMC C.C. Chen Institute of Health, West China School of Public Health and West China Fourth Hospital, Sichuan University Chengdu China; ^4^ School of Basic Medicine Southwest Medical University Luzhou China; ^5^ Department of Gastrointestinal Bariatric, and Metabolic Surgery Research Center for Nutrition Metabolism & Food Safety West China‐PUMC C.C. Chen Institute of Health West China School of Public Health and West China Fourth Hospital, Sichuan University Chengdu China; ^6^ Longgang Central Hospital Shenzhen China; ^7^ Department of Oncology and Department of Biomedical and Clinical Sciences Linköping University Linköping Sweden

**Keywords:** acute pancreatitis, CD320‐ablation mouse, Mendelian randomization (MR), vitamin B_12_

## Abstract

Pharmacological agents regarding the most optimal treatments of acute pancreatitis remain. One‐carbon metabolism nutrients as therapeutic agents in many diseases might be involved in acute pancreatitis. The roles are acquired exploration in acute pancreatitis. We utilized Mendelian randomization to assess the causal impact of folate, homocysteine, and vitamin B_12_ (VB_12_) on acute pancreatitis. Wild‐type and corresponding genetically modified mouse models were used to verify the genetic correlating findings. A negative association between genetically predicted serum VB_12_ levels and risks of acute pancreatitis was identified in human population. The transcobalamin receptor (TCblR)/*CD320* gene ablation that decreased cellular VB_12_ uptake and ATP production in pancreatic tissues promoted necrosis, resulting in much severe pathological changes of induced acute pancreatitis in mice. VB_12_ pretreatment and posttreatment dramatically increased ATP levels in pancreatic tissues and reduced the necrosis, then the elevated levels of amylase in serum, the levels of CK‐19, the activity of trypsin, and T lymphocyte infiltration in pancreatic tissues, prevented the pancreatic gross loss and ameliorated histopathological changes of mouse pancreases with induced acute pancreatitis. The results reveal that VB_12_ is potential as a therapeutic agent to inhibit tissue injuries and adaptive inflammatory responses in the pancreas in patients with acute pancreatitis.

## Graphical abstract

Combined with Mendelian randomization analysis and acute pancreatitis mouse models, this study uncovered that vitamin B_12_ prevents necrosis of pancreatic acinar cells in the progression of acute pancreatitis via ATP pathway. This finding offers a promising and accessible therapeutic option for acute pancreatitis.

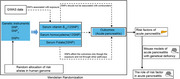

## 1 INTRODUCTION

Acute pancreatitis, characterized by acinar cell necrosis and extensive local and systemic inflammation, is a prevalent inflammatory disorder of the pancreas, resulting in significant morbidity and mortality and making it one of the primary gastrointestinal reasons for hospitalization.^1^, ^2^, ^3^ Most patients present with self‐limiting mild acute pancreatitis. Around 20% of patients suffer from moderate or severe acute pancreatitis, characterized by necrosis of the pancreatic or peripancreatic tissue, possibly accompanied by organ failure.^1^, ^2^ A substantial mortality rate of severe acute pancreatitis is about 20%‒40%.

During the first 1−2 weeks of acute pancreatitis development, acinar cells are the initial cell types to be injured in most forms of acute pancreatitis. The injured pancreatic acinar cells trigger a sterile proinflammatory response, usually resulting in systemic inflammatory response syndrome (SIRS)^2^, ^4^ through the production of cytokines. If the SIRS is severe, the proinflammatory mediators can induce early multiple‐organ failure. Up to date, many questions regarding the most optimal treatment of acute pancreatitis remain. Most importantly, pharmacological agents or strategies to inhibit the early organ injuries of systemic inflammatory responses in the pancreas and prevent subsequent organ failure in patients are required.

There is growing evidence to suggest that high‐dose parenteral vitamin B_12_ (VB_12_) may modulate inflammatory responses in multiple organs and even SIRS in critically ill patients,^5^, ^6^ such as septic shock^7^ and acute kidney injury.^8^ Recently, studies in vitro and in animals have suggested that imbalances in one‐carbon metabolism nutrients (vitamin B_6_, VB_12_, homocysteine, and folate) can negatively impact pancreatic cellular differentiation and the integrity of the intestinal mucosal barrier, exacerbating toxic damage and inflammatory responses.^9^, ^10^, ^11^, ^12^ Homocysteine stands as a pivotal metabolite that bridges the methylation, remethylation, and transsulfuration pathways, serving as the substrate for the biosynthesis of methionine through the catalytic action of VB_12_‐ and folate‐dependent methionine synthase or betaine‐homocysteine methyltransferase.^13^, ^14^ Two previous observational studies involving 14 and 20 patients, respectively, revealed that acute pancreatic patients have higher plasma homocysteine levels than healthy subjects, whereas levels of VB_12_ and folate appeared unaffected.^15^, ^16^ In a mouse model of acute pancreatitis, VB_12_ has been shown to stimulates clearance of reactive oxygen species by conserving glutathione (GSH) to protect pancreatic damage.^11^ These observations suggest that the one‐carbon metabolism nutrients might be involved in acute pancreatitis.

In the present study, we employed Mendelian randomization (MR) analysis using single nucleotide polymorphisms (SNPs) as an instrumental variables to assess potential causal links between key one‐carbon metabolism nutrients and the risk occurrence of acute pancreatitis. Furthermore, mouse models are used to verify the genetic correlating findings and deepen our understanding of the role of folate, homocysteine, and VB_12_ in acute pancreatitis, thereby offering valuable insights for future clinical treatments.

## 2 RESULTS

### 2.1 Genetically predicted serum VB_12_ levels, but not folate or homocysteine show a protective effect against the risk of acute pancreatitis in patients

**FIGURE 1:**
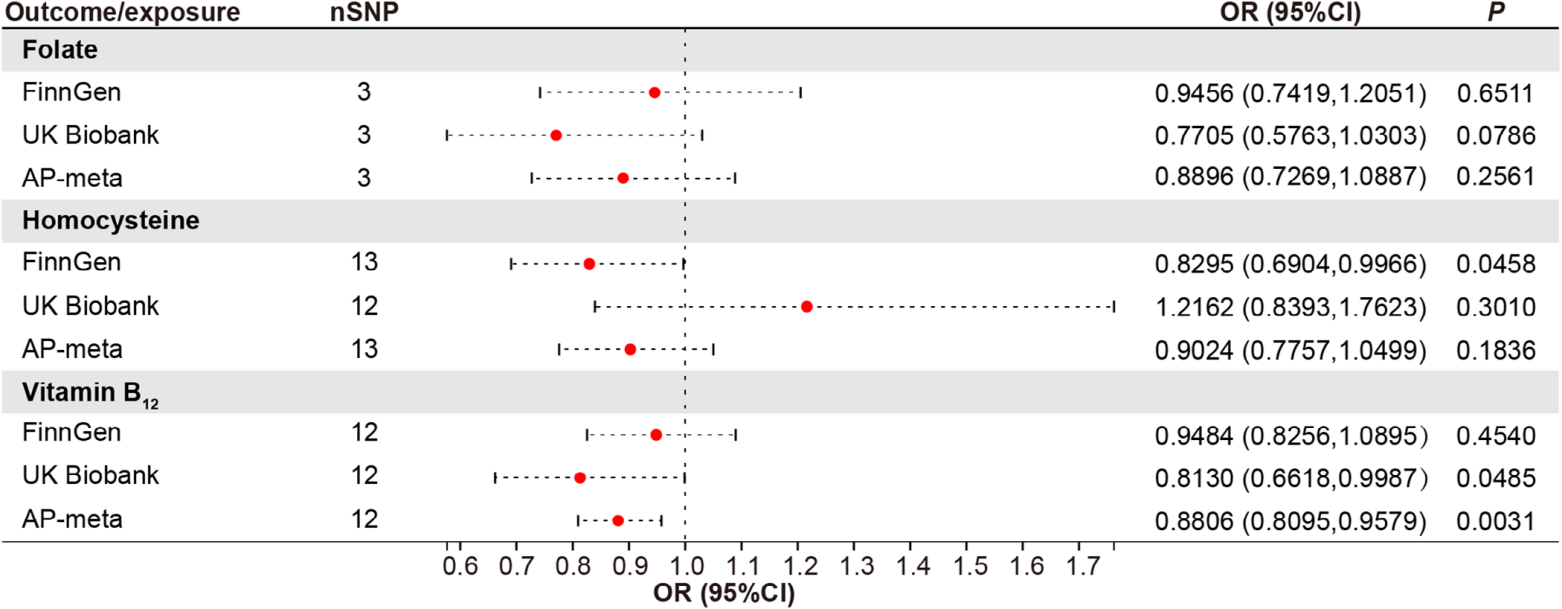
Mendelian randomization (MR) estimates for the association between folate, homocysteine, and vitamin B_12_ (VB_12_) with acute pancreatitis. Odds ratio (OR) was estimated using the random‐effects inverse variance weighted method. CI, confidence interval; SNPs, single‐nucleotide polymorphisms.

To test for the causal effect of key circulating one‐carbon metabolism nutrients on the risk of acute pancreatitis, 3, 13, and 12 SNPs robustly associated with serum folate, homocysteine, and VB_12_ levels, respectively, were used as instrumental variables (Table S2) to determine their effects on patients with acute pancreatitis. The MR results showed that the effect of homocysteine on acute pancreatitis was inconsistent in the FinnGen consortium (odds ratio [OR] = 0.8295, 95% confidence interval [CI] = 0.6904‒0.9966, *p *= 0.0458) and the UK Biobank cohort (OR = 1.2162, 95% CI = 0.8393‒1.7623, *p* = 0.3010) (Figure 1). The association between folate and acute pancreatitis was not observed (Figure 1). In contrast, the genetic prediction of the serum concentration of VB_12_ was causally associated with decreased risk of acute pancreatitis in the UK Biobank cohorts (OR = 0.8130, 95% CI = 0.6618‒0.9987, *p* = 0.0485) and consistently observed in the FinnGen consortium (OR = 0.9484, 95% CI = 0.8256‒1.0895, *p* = 0.4540). To derive comprehensive genome‐wide association study (GWAS) statistics for acute pancreatitis outcomes, we conducted a meta‐analysis using data from both the FinnGen consortium and the UK Biobank cohorts. This combined analysis revealed no significant correlations between the risks of acute pancreatitis and the genetically predicted serum levels of homocysteine or folate (Figure 1). A suggestively negative causal association between serum VB_12_ levels and the risks of acute pancreatitis in the human population was detected (Figure 1). For one‐unit or one‐standard deviation (SD) increase in the prevalence of these traits, the combined OR and corresponding 95% CI were 0.8806 (95% CI = 0.8095 ‒0.9579, *p* = 0.0031) for acute pancreatitis. The results remained directionally consistent in MR‐Egger and weighted median model (Table 1).

**TABLE 1:** Associations of genetically predicted risks factors with acute pancreatitis in sensitivity analysis.

	Weighted median			MR‐Egger				MR‐Egger, Cochran's *Q*			IVW, Cochran's *Q*			MR‐PRESSO	
Exposure/outcome	OR	95% CI	*p*‐value	OR	95% CI	*p*‐value	*P*_intercept_	*Q*	*I* ^2^	*P*_Q	*Q*	*I* ^2^	*P*_Q	Causal estimate	*P*_Glo
Folate															
FinnGen	0.961	0.6359, 1.4522	0.850	1.332	0.5102, 3.4789	0.663	0.580	0.076	0	0.783	0.679	0	0.712	NA	NA
UK Biobank	0.774	0.3881, 1.5420	0.466	0.779	0.1719, 3.5344	0.801	0.990	0.402	0	0.526	0.403	0	0.818	NA	NA
AP‐meta	0.896	0.6296, 1.2752	0.542	1.133	0.4965, 2.5835	0.817	0.639	0.237	0	0.626	0.642	0	0.725	NA	NA
Homocysteine															
FinnGen	0.972	0.7662, 1.2321	0.812	1.168	0.7983, 1.7072	0.442	0.073	9.444	0	0.581	13.373	0.10	0.343	‒0.187	0.371
UK Biobank	1.162	0.8095, 1.6675	0.416	0.713	0.3238, 1.5688	0.420	0.137	17.117	0.36	0.105	21.131	0.43	0.049	0.210	0.071
AP‐meta	0.917	0.7514, 1.1180	0.390	1.088	0.7815, 1.5144	0.628	0.239	10.442	0	0.491	11.993	0	0.446	‒0.103	0.506
VB_12_															
FinnGen	0.888	0.7421, 1.0625	0.194	0.700	0.5259, 0.9311	0.034	0.041	6.693	0	0.754	12.217	0.10	0.348	‒0.053	0.372
UK Biobank	0.833	0.6690, 1.0366	0.101	0.842	0.5914, 1.1988	0.363	0.866	17.259	0.36	0.101	17.306	0.31	0.139	‒0.204	0.220
AP‐meta	0.874	0.7610, 1.0033	0.056	0.777	0.6293, 0.9599	0.041	0.210	5.120	0	0.883	6.918	0	0.806	‒0.127	0.813

*Note*: The ORs of AP were scaled to one‐SD increase in folate, homocysteine, and VB_12_, one‐unit increase in OR of AP. *p*‐Values for pleiotropy were derived from MR‐Egger test and a *p*‐value < 0.05 indicates a possible pleiotropic effect. *p*‐Values for distortion were derived from MR‐PRESSO test and a *p*‐value < 0.05 indicates a difference between estimates before and after outlier removal.

Abbreviations: AP, acute pancreatitis; AP‐meta, combination of data from the FinnGen consortium and the UK Biobank cohort; CI, confidence interval; IVW, inverse variance weighted; MR, Mendelian randomization; MR‐PRESSO, Mendelian randomization Pleiotropy Residual Sum and Outlier; NA, not available; OR, odds ratio; *P*_Glo, *p*‐value for global test; *P*_Q, *p*‐value for Q test; *P*_intercept_, *p*‐value for the intercept in the MR‐Egger regression was used present the pleiotropy; SD, standard deviation; VB_12_, vitamin B_12_.

Small heterogeneity was noticed in the analyses of acute pancreatitis (Figure S1). In the sensitivity analysis with each SNP that genetically predicted VB_12_ levels, the associations remained (Table 1). Additionally, leave‐one‐out analyses showed that no single SNP drove the estimates (Figure S2), confirming the reliability of the causal effects of VB_12_ levels in serum on acute pancreatitis in humans. Meanwhile, MR‐Egger and MR‐PRESSO tests showed no evidence of horizontal pleiotropy (Table 1). These results demonstrated that genetically predicted serum VB_12_ levels are negatively associated with the risks of acute pancreatitis in patients.

### 2.2 Acute pancreatitis is aggravated in *CD320*‐ablation mouse models

**FIGURE 2:**
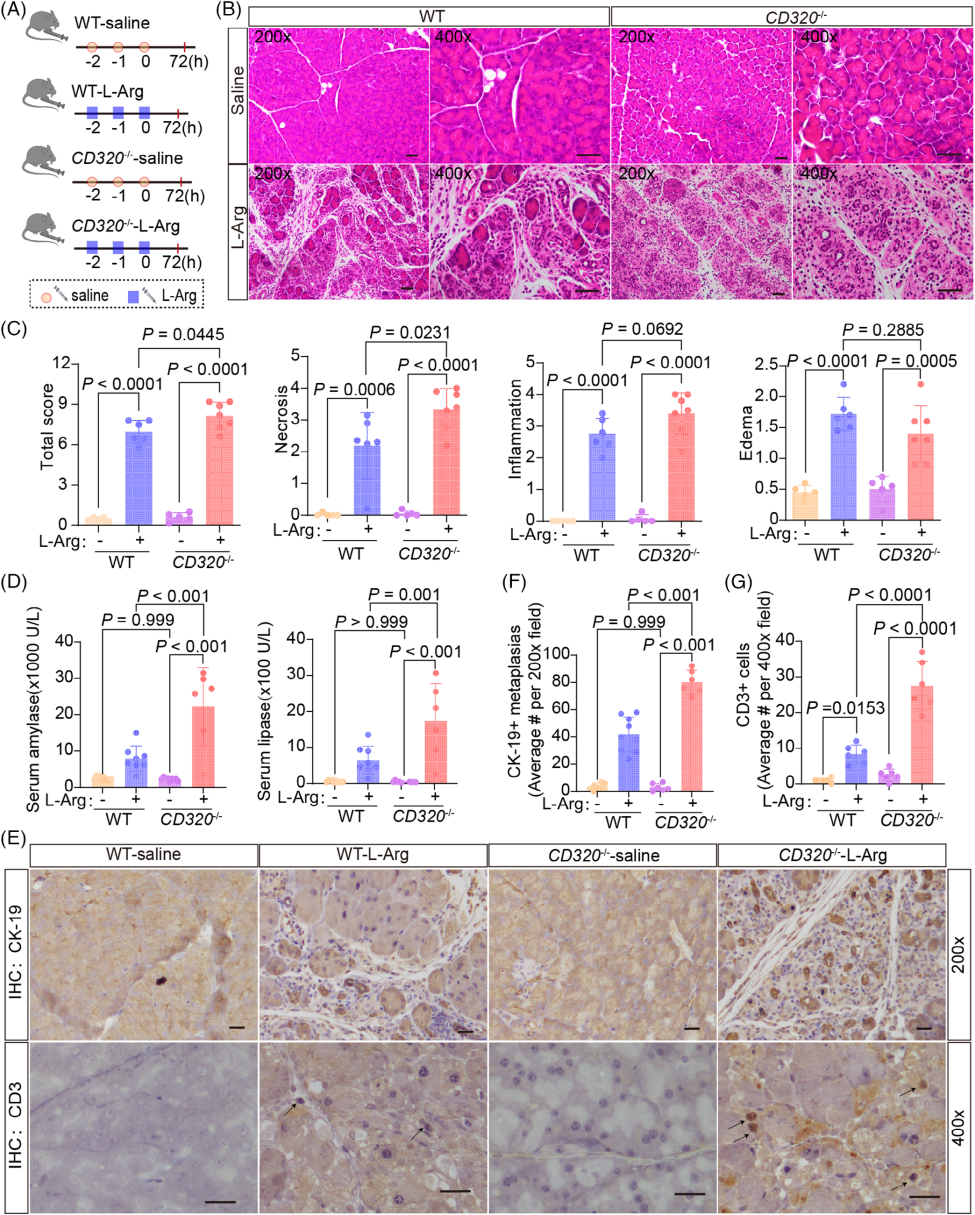
Acute pancreatitis induced by L‐Arg was aggravated in *CD320*‐ablation mice. (A) The flow charts of experiment. (B) The representative histological images of the pancreas were obtained 72 h after the final administration of L‐Arg (scale bar, 50 µm). (C) The histological evaluation of pancreatic edema, inflammatory infiltration, necrosis, and the overall sum of these features. (D) Serum amylase and lipase levels were measured at 72 h after the final administration of L‐Arg. (E) The representative immunohistochemical images for CK‐19 and CD3+ T lymphocyte cell of mouse pancreatic tissues at 72 h after the final administration of L‐Arg (scale bar, 50 µm). (F and G). Quantification of immunohistochemical analysis for CK‐19 and CD3+ T lymphocyte cell of mouse pancreatic tissues at 72 h after the final administration of L‐Arg, respectively. The data were obtained from 5−10 mice per group and presented as mean ± standard deviation (SD) from individual mice with one‐way analysis of variance (ANOVA) and Tukey's multiple‐comparison posttest.

Although the MR analysis provided an associated relationship between serum VB_12_ levels and acute pancreatitis, the roles of VB_12_ in the progress of acute pancreatitis remained to be directly elucidated. Therefore, a genetically modified mouse model involving the ablation of the *CD320* gene was used to evaluate the effects of cobalamin homeostasis on acute pancreatitis (Figure S3A‒D). The ablation of the *CD320* gene causes the defects of cellular VB_12_ uptake in cells in mouse tissues,^17^ resulting in reduced VB_12_ levels in pancreatic tissues of mice (Figure S3E). Intraperitoneal L‐arginine (L‐Arg) administration is widely used to induce necrotizing pancreatitis in animal models. This non‐invasive model leads to targeted, dose‐responsive necrosis in acinar cell, making it an effective model for exploring the pathological mechanisms of acute necrotizing pancreatitis as well as the temporal evolution of the disease. Thus, an acute pancreatitis mouse model was generated by administering with L‐Arg (Figure 2A). Seventy‐two hours after L‐Arg treatment, the mice were examined. The level of VB_12_ in serum and pancreatic tissue significantly decreased in wild‐type mice, but not *CD320*‐ablation mice after administering with L‐Arg (Figure S4A). In the wild‐type mice, majorities of the mouse pancreatic lobules turned loose, pancreatic edema, necrosis, inflammatory cell infiltrations, and acinar atrophy were also observed (Figure 2B,C). Levels of amylase and lipase in serum were significantly increased at the 72 h stage of the disease (Figure 2D). The ductal tubular complex is an important pathological manifestation of acinar cell death in acute pancreatitis and can be recognized by CK‐19, a sensitive duct marker.^18^ As acute pancreatitis progressed, many pancreatic epithelial cells changed to a flat shape and formed tube‐like structures with strong staining of CK‐19 at the 72 h stage of the disease (Figure 2E,F). In *CD320*‐ablation mice, acute pancreatitis induced with L‐Arg produced much severer pancreatic histopathological changes (Figure 2B,C). The *CD320*‐ablation greatly increased the levels of amylase and lipase in serum (Figure 2D) and CD3^+^ T lymphocyte infiltration (Figure 2E,G), as well as the formation of ductal tubular complexes (Figure 2E,F) in the pancreas in mice with L‐Arg‐induced acute pancreatitis. *CD320*‐ablation did not increase or slightly increased the level of activity of trypsin, CD11b^+^ macrophages, and MPO^+^ neutrophil infiltrations in pancreatic tissues (Figure S4B‒F). The results suggest that the VB_12_ deficiency in cells can enhance acinar cell injuries, promote the formation of ductal tubular complexes, and trigger T lymphocyte infiltration in L‐Arg‐induced acute pancreatitis in mice.

**FIGURE 3:**
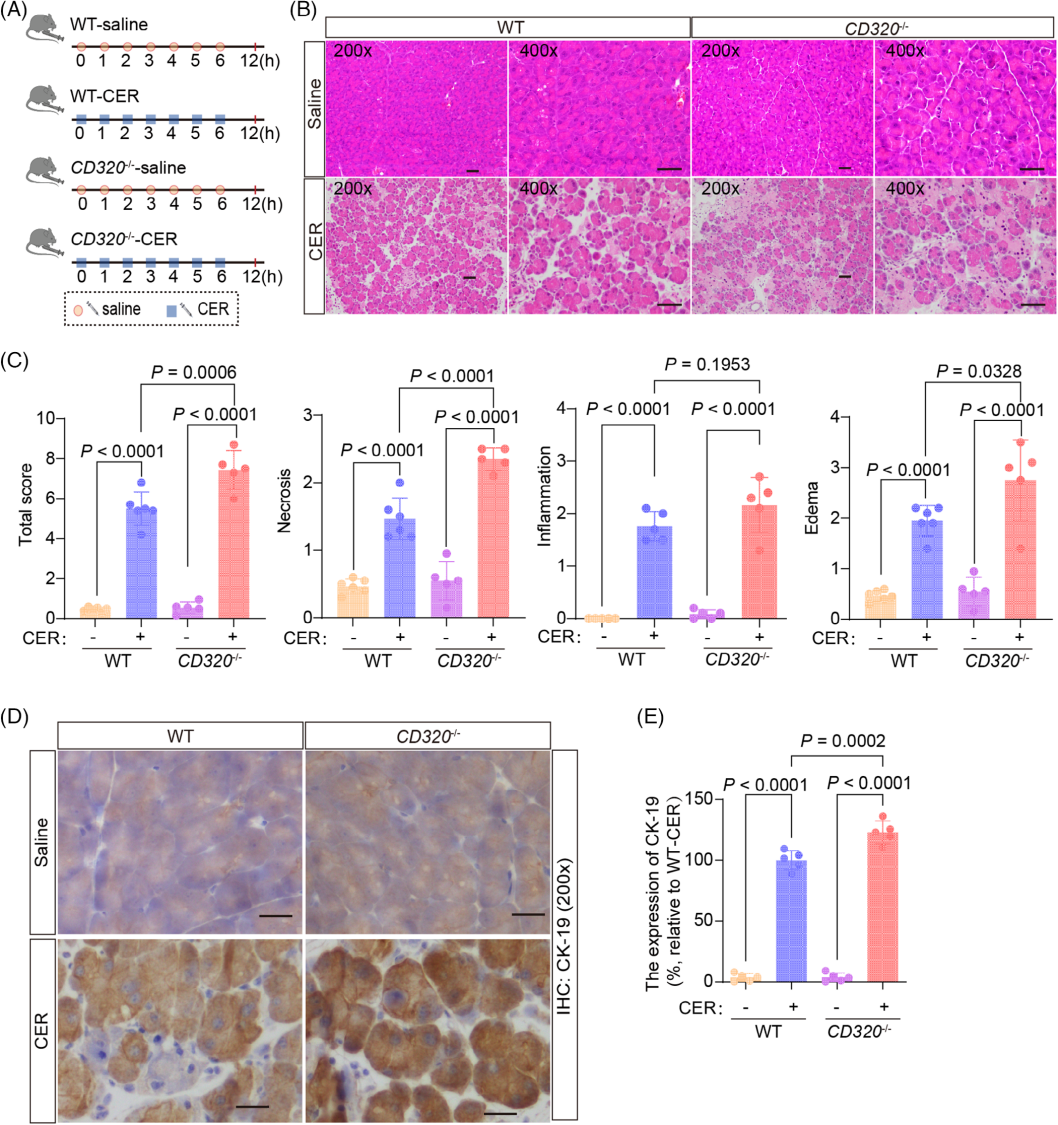
Acute pancreatitis induced by cerulein (CER) was aggravated in *CD320*‐ablation mice. (A) The flow charts of experiment. (B) The representative histological images of the pancreas were obtained 12 h after the first administration of CER (scale bar, 50 µm). (C) The histological evaluation of pancreatic edema, inflammatory infiltration, necrosis, and the overall sum of these features. (D) The representative immunohistochemical images for CK‐19 of mouse pancreatic tissues at 12 h after the first administration of CER (scale bar, 50 µm). (E) Quantification of immunohistochemical analysis for CK‐19 of mouse pancreatic tissues at 12 h after the first administration of CER. The data were obtained from five mice per group and presented as mean ± standard deviation (SD) from individual mice with one‐way analysis of variance (ANOVA) and Tukey's multiple‐comparison posttest.

Acute pancreatitis induced by cerulein (CER), a cholecystokinin analog, was the most well‐characterized and widely used experimental model for acute edematous pancreatitis.^19^, ^20^ During the initial stages of pancreatitis triggered by CER, the fusion of zymogen granules within acinar cells gives rise to prominent autophagic vacuoles. This process is coupled with a surge in lysosomal enzyme activity and activation of trypsinogen, ultimately culminating in cellular necrosis.^19^, ^21^ In the CER model, the pancreas begins to recover 12 h after induction of pancreatitis.^22^ This model of CER‐induced pancreatitis favors the analysis of intracellular events in the early phase of pancreatitis and can be used to define the early phenotypes occurring in pancreatic tissues in mice. After administering CER in wild‐type and *CD320*‐ablation mice (Figure 3A), the level of VB_12_ in serum and pancreatic tissues significantly decreased in wild‐type mice, but not in *CD320*‐ablation mice (Figure S5A), which is consistent with the L‐Arg‐induced acute pancreatitis models. CER induced much severer pancreatic histopathological changes with increased necrosis and ductal tubular complexes in *CD320*‐ablation mice compared to wild‐type mice (Figure 3B‒E). The levels of amylase and lipase in serum (Figure S5B), the trypsin activities (Figure S5C), and MPO^+^ neutrophil infiltration (Figure S5D) in pancreatic tissues did not change or slightly increased in *CD320*‐ablation mice, in comparison to the phenotypes in wild‐type mice. In addition, significant CD3^+^ T lymphocyte infiltration was not detected in pancreatic tissues in the early phase of the CER‐induced acute pancreatitis models (data not shown). The experiments of CER‐induced acute pancreatitis models indicate that VB_12_ directly protects necrosis of acinar cells during the early stage of acute pancreatitis. T lymphocyte infiltration most likely was secondary effects after the necrosis of acinar cells in L‐Arg‐induced acute pancreatitis in mice.

### 2.3 Vitamin B_12_ displays therapeutic effects on acute pancreatitis in mouse models

**FIGURE 4:**
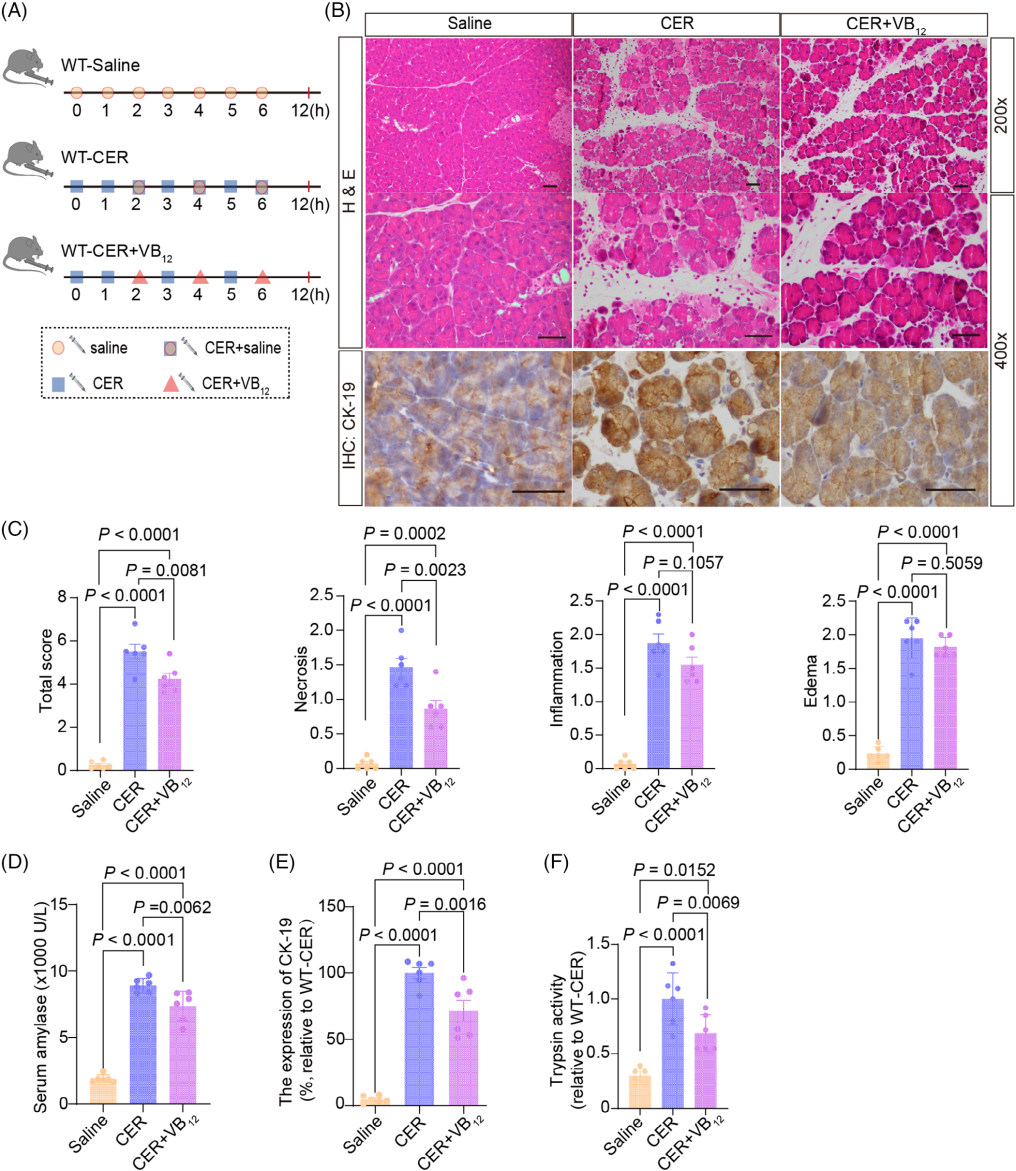
Vitamin B_12_ (VB_12_) exhibits therapeutic effects on experimental acute pancreatitis induced by cerulein (CER). (A) The flow charts of experiment. (B) The representative histological and immunohistochemical images of the pancreas for CK‐19 were obtained 12 h after the first administration of CER (scale bar, 50 µm). (C) The histological evaluation of pancreatic edema, inflammatory infiltration, necrosis, and the overall sum of these features. (D) Serum amylase levels were measured at 12 h after the first administration of CER. (E). Quantification of immunohistochemical analysis for CK‐19 of mouse pancreatic tissues at 12 h after the first administration of CER. (F) Representative of trypsin activity in pancreatic tissues at 12 h after the first administration of CER. The data were obtained from five to six mice per group and presented as mean ± standard deviation (SD) from individual mice with one‐way analysis of variance (ANOVA) and Tukey's multiple‐comparison posttest.

We used CER‐induced pancreatitis to test the effects of VB_12_ on the intracellular events in pancreatic tissues in wild‐type mice (Figure 4A). After treatment with VB_12_, the level of VB_12_ in pancreatic tissues significantly increased (Figure S6A). The pancreatic necrosis (Figure 4B,C), the level of serum amylase (Figure 4D), the formation of ductal tubular complexes (Figure 4B,E), and the trypsin activities (Figure 4F) in pancreatic tissues in mice with CER‐induced acute pancreatitis significantly reduced. In contrast, the VB_12_ treatment was unable to reduce the level of serum lipase (Figure S6B), the MPO^+^ neutrophil infiltration (Figure S6C) and CD11b^+^ macrophage (Figure S6D,E) in pancreatic tissues in mice with CER‐induced acute pancreatitis. In addition, we did not detect significant CD3^+^ T lymphocyte infiltration (Figure S6D,F) in pancreatic tissues in the early phase of the CER‐induced acute pancreatitis models. The results confirm that VB_12_ can restore the pancreatic structures and prevent acinar cell necrosis in the early phase of pancreatitis in mouse models with CER‐induced pancreatitis and is unable to prevent the innate immune responses in pancreatic tissues with induced acute pancreatitis in mice.

**FIGURE 5:**
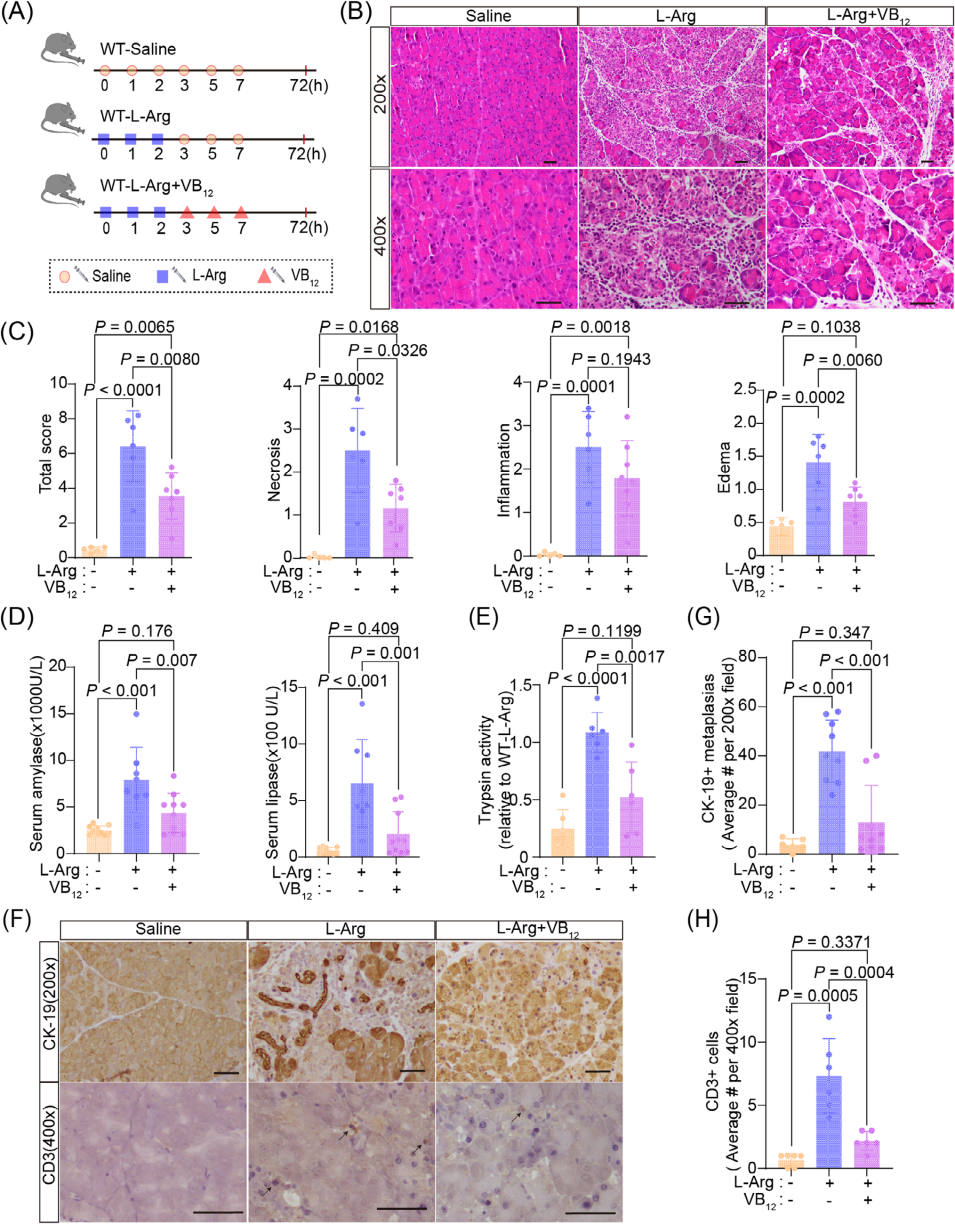
Vitamin B_12_ (VB_12_) exhibits therapeutic effects on experimental acute pancreatitis induced by L‐Arg. (A) The flow charts of experiment. (B) The representative histological images of pancreas were obtained 72 h after the final administration of L‐Arg (scale bar, 50 µm). (C) The histological evaluation of pancreatic edema, inflammatory infiltration, necrosis, and the overall sum of these features. (D) Serum amylase and lipase levels were measured at 72 h after the final administration of L‐Arg. (E) Representative of trypsin activity in pancreatic tissues at 72 h after the final administration of L‐Arg. (F) The representative immunohistochemical images for CK‐19 and CD3+ T lymphocyte cell of mouse pancreatic tissues at 72 h after the final administration of L‐Arg (scale bar, 50 µm). (G and H) Quantification of immunohistochemical analysis for CK‐19 and CD3+ T lymphocyte cell of mouse pancreatic tissues at 72 h after the final administration of L‐Arg, respectively. The data were obtained from 5−10 mice per group and presented as mean ± standard deviation (SD) from individual mice with one‐way analysis of variance (ANOVA) and Tukey's multiple‐comparison posttest.

Next, we tested the effect of VB_12_ in L‐Arg‐induced acute pancreatitis model (Figure 5A). VB_12_ treatment greatly reduced the pancreatic histopathological changes of acute pancreatitis in mouse models (Figure 5B,C). The levels of amylase and lipase in serum (Figure 5D), trypsin activities (Figure 5E), ductal tubular complexes (Figure 5F,G), CD3^+^ T lymphocyte infiltration (Figure 5F,H), and CD11b^+^ macrophage (Figure S7B,C) in pancreatic tissue with L‐Arg‐induced acute pancreatitis were dramatically reduced by VB_12_ treatment. Furthermore, a notable decrease in the infiltration of CD8^+^ T lymphocyte, rather than CD4^+^ cell, was observed, accompanied by an increase in Treg cell in CD4^+^ T lymphocyte (Figure S7E). The results show that VB_12_ plays a protective role in L‐Arg‐induced pancreatitis in mice.

**FIGURE 6:**
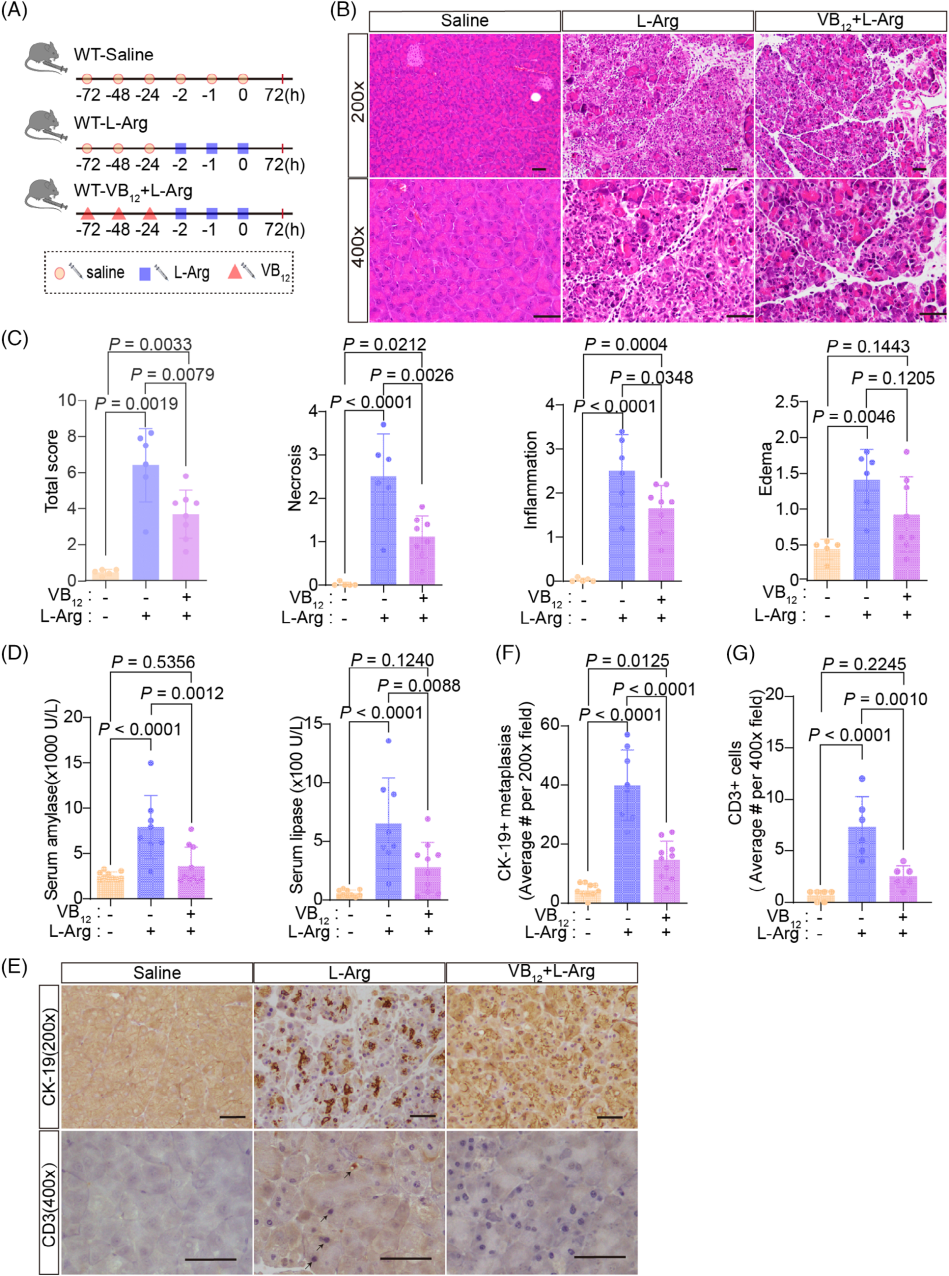
Vitamin B_12_ (VB_12_) displays preventive effects on acute pancreatitis induced by L‐Arg in mouse models. (A) The flow charts of experiment. (B) The representative histological images of pancreas were obtained 72 h after the final administration of L‐Arg (scale bar, 50 µm). (C) The histological evaluation of pancreatic edema, inflammatory infiltration, necrosis, and the overall sum of these features. (D) The levels of serum amylase and lipase were measured at 72 h after the final administration of L‐Arg. (E) The representative immunohistochemical images for CK‐19 and CD3+ T lymphocyte cell of mouse pancreatic tissues at 72 h after the final administration of L‐Arg (scale bar, 50 µm). (F and G) Quantification of immunohistochemical analysis for CK‐19 and CD3+ T lymphocyte cell of mouse pancreatic tissues at 72 h after the final administration of L‐Arg, respectively. The data were obtained from 5−10 mice per group and presented as mean ± standard deviation (SD) from individual mice with one‐way analysis of variance (ANOVA) and Tukey's multiple‐comparison posttest.

To test whether VB_12_ conducts prevention of occurrences of acute pancreatitis (Figure 6A). VB_12_ pretreatment prevented the gross loss of pancreatic structures, ameliorated pancreatic histopathological changes (Figure 6B,C), and dramatically reduced the elevation of amylase levels in serum (Figure 6D), the formation of ductal tubular complexes (Figure 6E,F), CD3^+^ T lymphocyte infiltration (Figure 6E,G), the CD45^+^ immune cell (Figure S6C,E), and the activity of trypsin in the pancreatic tissues (Figure S8A) in mice with induced acute pancreatitis. Furthermore, there was a notable increase in the proportion of Treg cell in CD4^+^ T lymphocyte (Figure S8F). CD11b^+^ macrophage and MPO^+^ neutrophil infiltrations were not altered in pancreatic tissues (Figure S8B‒D). These results demonstrate that VB_12_ pretreatment can prevent injuries of acinar cells and reduces inflammatory reactions of T lymphocytes in pancreatic tissues in mice with L‐Arg‐induced acute pancreatitis.

### 2.4 Vitamin B_12_ protects the acinar cells via ATP in pancreatic tissues in mouse models

VB_12_ participates in two biochemical reactions: the conversion of homocysteine to methionine, and *N* ^5^‐methyl‐tetrahydrofolate to tetrahydrofolate by the cytosolic methionine synthase, which requires methyl‐Cbl as a cofactor and the conversion of methyl malonyl‐coenzyme A to succinyl‐CoA, the molecule is mostly involved in tricarboxylic acid cycle for ATP production.^23^ Genetic analysis showed that a high level of circulation VB_12_, but not folate and homocysteine, reduced the risk of acute pancreatitis, excluding the possibility that VB_12_ displayed a protective role in the progress of acute pancreatitis via homocysteine or folate pathways. Homocysteine is involved in oxidative stress pathways and VB_12_ has been shown to conserve GSH to reduce oxidative stress in pancreatic tissues in a mouse model with acute pancreatitis. We subjected GSH into the *CD320*‐ablation mice and induced acute pancreatitis by CER (Figure S9A). The results showed that GSH enhanced the necrosis in pancreatic tissues in the mouse models (Figure S9B‒D). The tests exclude that VB_12_ clears reactive oxygen species by conserving GSH to protect pancreatic damage in pancreatic tissues of acute pancreatitis.

Most likely, VB_12_ restores the pancreatic structures and protects the acinar cells in the early phase of pancreatitis in mouse models via succinyl‐CoA‐based energy metabolism, which is involved in ATP production. Indeed, the level of ATP was greatly reduced in pancreatic tissues in *CD320*‐ablation mice (Figure S10A). After administering L‐Arg or CER, the ATP greatly lose in pancreatic tissues in wild‐type mice and *CD320*‐ablation mice (Figure S10A). However, the decreased degree of ATP levels was smaller in the pancreatic tissues of *CD320*‐ablation mice, in comparison to the ones in wild‐type mice (Figure S10B). The phenotypes are consistent with reduced VB_12_ in pancreatic tissues in wild‐type mice and *CD320*‐ablation mice after induction of acute pancreatitis. After pretreatment or treatment with VB_12_ in mouse models, the level of ATP significantly increased in pancreatic tissues (Figure S10C).

**FIGURE 7:**
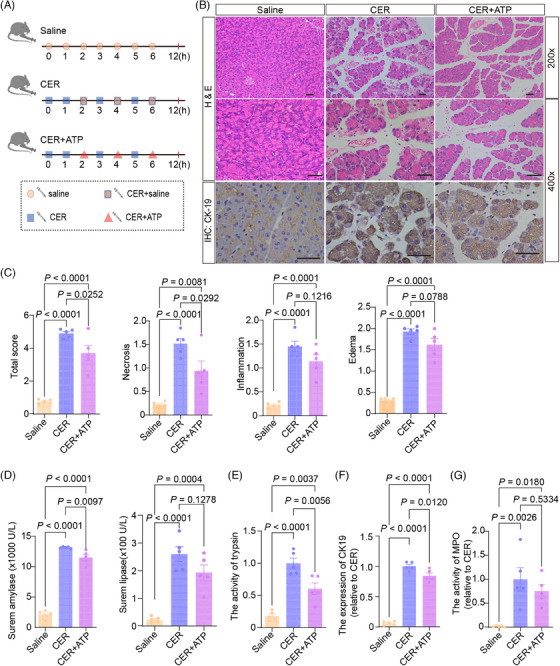
Vitamin B_12_ (VB_12_) protects the acinar cells via ATP production in pancreatic tissues in *CD320*‐ablation mice. (A) The flow charts of experiment. (B) The representative histological and immunohistochemical images of the pancreas for CK‐19 were obtained 12 h after the first administration of cerulein (CER) (scale bar, 50 µm). (C) The histological evaluation of pancreatic edema, inflammatory infiltration, necrosis, and the overall sum of these features. (D) The levels of serum amylase and lipase were measured at 12 h after the first administration of CER. (E) Representative of trypsin activity in pancreatic tissues at 12 h after the first administration of CER. (F) Quantification of immunohistochemical analysis for CK‐19 of mouse pancreatic tissues at 12 h after the first administration of CER. (G) Representative of activity of MPO in pancreatic tissue at 12 h after the first administration of CER. The data were obtained from 5−10 mice per group and presented as mean ± standard deviation (SD) from individual mice with one‐way analysis of variance (ANOVA) and Tukey's multiple‐comparison posttest.

To further verify the results, ATP was used to treat *CD320*‐ablation mice with CER‐induced acute pancreatitis (Figure 7A). As expected, the ATP treatment greatly reduced the pancreatic histopathological changes of acute pancreatitis in *CD320*‐ablation mouse models (Figure 7B,C). ATP treatment dramatically reduced the cell necrosis, the ductal tubular complexes, trypsin activities in the pancreatic tissue, and the serum amylase levels in the *CD320*‐ablation mouse models with CER‐induced acute pancreatitis (Figure 7B‒F). In contrast, the serum lipase levels and the MPO^+^ neutrophil infiltration in pancreatic tissues did not change after ATP treatment in the *CD320*‐ablation mouse models with CER‐induced acute pancreatitis (Figure 7D,G). The results that ATP treatment was able significantly to reduce the cell necrosis, but not the ductal tubular complexes, trypsin activities in the pancreatic tissues and the serum amylase levels in the wild‐type mouse models with CER‐induced acute pancreatitis further verified that ATP protected the cellular necrosis in pancreatic tissues with acute pancreatitis (Figure S11A‒G). Thus, the results indicate that VB_12_ restores the pancreatic structures and protects the acinar cells in the early phase of pancreatitis in mouse models via the ATP production pathway.

## 3 DISCUSSION

Here, we observed that a high level of circulation VB_12_, but not folate and homocysteine, reduces the risk of acute pancreatitis, suggesting a protective role of VB_12_ in the progress of acute pancreatitis. Furthermore, we showed that after induction of acute pancreatitis, VB_12_ in serum and pancreatic tissues was significantly reduces in wild‐type mice, suggesting VB_12_ is required in the pathological processes of acute pancreatitis. Indeed, VB_12_ uptake deficiency in pancreatic cells directly promotes acinar cell necrosis and the formation of ductal tubular complexes and does not play a role in the innate immune responses and T lymphocyte infiltration in mouse pancreatic tissues with induced acute pancreatitis. VB_12_ can be used as a preventive and therapeutic agent to restore the pancreatic structures and to reduce the inflammatory response, especially the inflammatory response of T lymphocytes in the pancreas of mouse models with induced acute pancreatitis. Interestingly, in the L‐arginine‐induced acute pancreatitis model, VB_12_ intervention attenuated acute pancreatitis while simultaneously increasing the proportion of Treg cell subtypes in CD4^+^ T lymphocyte. the mechanisms underlying how VB_12_ regulates T‐cell subtypes and its role in acute pancreatitis remain to be further explored.

To our knowledge, the results are the first one to evaluate the causal relationships between VB_12_ and acute pancreatitis risk and provides evidence to show the preventive and therapeutic potentials of VB_12_ in the treatment of acute pancreatitis.VB_12_, a crucial water‐soluble micronutrient, is obtained primarily through the consumption of foods, such as fish, meat, fortified cereals, and supplements. It is co‐absorbed along with intrinsic factors secreted by the parietal cells of the stomach in the terminal ileum.^24^ VB_12_ plays a vital role in two biochemical reactions. First, it facilitates the conversion of homocysteine to methionine, and *N* ^5^‐methyl‐tetrahydrofolate to tetrahydrofolate by the cytosolic methionine synthase, which requires methyl‐Cbl as a cofactor. Second, VB12 is involved in the transformation of methyl malonyl‐coenzyme A to succinyl‐CoA, catalyzed by the methyl malonyl‐CoA mutase in the mitochondria. This process utilizes 5′‐deoxy adenosyl‐Cbl as its cofactor.^25^ Our MR analysis results indicate that folate and homocysteine are not associated with acute pancreatitis in humans, excluding the possibility that VB_12_ displays a protective role in the progress of acute pancreatitis via homocysteine or folate pathway. Most likely, VB_12_ restores the pancreatic structures and protects the acinar cells in the early phase of pancreatitis in mouse models via succinyl‐CoA‐based energy metabolism involved in ATP production. Extracellular ATP acts as a damage‐associated molecular pattern, signaling tissue damage or cellular stress to the immune system. It is released by cells under stress or damage and may activate the inflammasome, eliciting the production of proinflammatory cytokines, including IL‐1β and IL‐18.^26^ However, the role of ATP is dependent on specific conditions; for instance, in low concentrations or with prolonged exposure, ATP may exhibit anti‐inflammatory properties. Extracellular purines can modulate immune responses, balancing inflammatory processes and immunosuppression.^27^ During acute pancreatitis, acinar cells undergo edema, leading to an increase in cell permeability, which in turn enhances the absorption of extracellular ATP. Besides, As reported in a study, extracellular ATP is internalized by macropinocytosis, leading to an increase in intracellular ATP levels.^28^, ^29^ Based on our findings, ATP supplementation did not significantly increase the level of inflammation but effectively prevent cell necrosis during the progresses of induced acute pancreatitis in both VB_12_ uptake deficiency mice and wild‐type mice. Thus, VB_12_ restores the pancreatic structures and protects the acinar cells in the early phase of pancreatitis in mouse models via the ATP production pathway.

In the United States and the UK, approximately 6% of individuals under 60 years old suffer from VB_12_ deficiency, and nearly 20% among those aged 60 and above.^30^ In Latin American nations, a staggering 40% of the population experiences either clinical or subclinical deficiency of VB_12_. Among Kenyan school children, the prevalence stands at 70%, while it reaches 80% in East Indian preschoolers and 70% in East Indian adults.^31^  The risk factors increased VB_12_ deficiency rate included dietary insufficiency, pernicious anemia, and prolonged use of medications such as metformin or acid suppressing.^30^, ^31^, ^32^, ^33^ Therefore, when the VB_12_ deficiency is determined, acute pancreatitis should be noticed beyond severe known complications including the degeneration of the spinal cord and pancytopenia in patients.^34^ VB_12_ deficiency should be managed appropriately to prevent severe acute pancreatitis.

Currently, therapeutic approaches for acute pancreatitis include fluid therapy, enteral nutrition, urgent endoscopic retrograde cholangiopancreatography (ERCP), and cholecystectomy within the first 72 h of acute pancreatitis. Administering fluid therapy helps to prevent hypovolemia and ensure adequate organ perfusion in patients with acute pancreatitis.^35^ Additionally, enteral nutrition plays a vital role in safeguarding the gut's mucosal barrier and minimizing bacterial translocation, potentially reducing the chances of infected pancreatic and peripancreatic necrosis.^36^ For patients diagnosed with acute cholangitis, urgent ERCP is recommended regardless of whether acute biliary pancreatitis is also present. In instances where acute pancreatitis arises as a complication of gallstone disease, cholecystectomy becomes necessary to prevent future occurrences of acute pancreatitis.^37^ All of these interventions represent best practices aimed at minimizing morbidity and mortality in acute pancreatitis. Currently, there is lack of pharmacological treatments specifically designed for acute pancreatitis. Nevertheless, a study has proposed that chenodeoxycholic acid as a promising approach for addressing acinar cell necrosis, a key pathological feature of acute pancreatitis.^9^ Compared to chenodeoxycholic acid, VB_12_ may be a preferred therapeutic choice for the treatment of acute pancreatitis, owing to its ease of access, superior safety profile, and the lack of significant adverse effects even at high dosages.^38^, ^39^ Now, we show that VB_12_, not homocysteine and folate, is negatively associated with the risk of acute pancreatitis. VB_12_ treatments can directly prevent the necrosis of acinar cells, subsequently reduce the inflammatory response, especially the inflammatory response of T lymphocytes in the pancreas during the occurrence of acute pancreatitis and displays therapeutic effects on acute pancreatitis in mouse models. Next, a clinical trial should be launched to test whether VB_12_ treatment, especially within the first 24 h of acute pancreatitis, reduce morbidity and mortality of acute pancreatitis in patients.

Several limitations should be acknowledged in our study. First, the study cohorts are predominantly from European populations, which restricts the applicability of our findings to ethnically diverse groups such as Asians and Africans. Second, the absence of individual‐level data prevents a comprehensive analysis of the relationship between VB_12_‐related genetic SNPs and various aspects of acute pancreatitis, including its severity, etiology (alcohol‐induced or gallstone‐related), and other clinical features. Third, the current study focuses mainly on the protective effect of VB_12_ on acinar cells in pancreatic tissue. However, complications of acute pancreatitis, such as pneumonia and infection, have not been monitored.

## 4 MATERIALS AND METHODS

### 4.1 Genetic instrument selection for exposure

The independent SNPs of folate,^40^ homocysteine,^41^ and VB_12_^40^ were identified from corresponding meta‐analyses of GWAS including 37,465, 44,147, and 45,576 individuals of European ancestries, respectively (Table S1). At the genome‐wide significance threshold of *p* < 5 × 10^−8^, we extracted those SNPs significantly associated with serum folate, homocysteine, and VB_12_ levels. To refine our selection, we excluded any SNPs that exhibited linkage disequilibrium, specifically those with an *r* ^2^ value below 0.001 within a 10‐Mb region. The *R* ^2^ and *F* statistic of each SNP was calculated using the formula: *R* ^2^ = 2 × MAF × (1 − MAF) × *β* ^2^, and *F* statistic = *R* ^2^ × (*N* − 2)/(1 − *R* ^2^). Subsequently, we excluded any SNPs with an *F* statistic fell below 10. We extracted 3, 14, and 14 SNPs robustly associated with serum folate, homocysteine, and VB_12_ as instrumental variables (Table S2). Three of these SNPs (rs7788053, rs1141321, and rs2251468) were absent from at least one of our acute pancreatitis cohorts. The final set of instrumental variables comprised 3, 13, and 12 genetic variants for serum folate, homocysteine, and VB_12_, respectively (Table S2).

### 4.2 Outcome data sources and meta‐analysis

We analyzed the GWAS summary statistics pertaining to acute pancreatitis, harnessing data from two prominent, publicly available biobank‐scale cohorts: the FinnGen consortium and the UK Biobank cohort (Table S1). FinnGen represents a comprehensive study that integrates both population‐based and disease‐oriented cohorts while the UK Biobank stands as a prospective, population‐centered initiative, focusing primarily on UK citizens between the ages of 40 and 69 years.^42^ Pre‐computed acute pancreatitis summary statistics of the FinnGen released 7 data was collected from the project website (https://r7.finngen.fi/). Acute pancreatitis GWAS summary statistics from fastGWA analysis of the UK Biobank imputed data were obtained from the website (https://yanglab.westlake.edu.cn/data/ukb_fastgwa/imp_binary/). The FinnGen consortium included 4648 cases of acute pancreatitis and 273,442 controls, while the UK Biobank cohort included 1748 cases of acute pancreatitis and 454,600 controls (Table S1). In this study, summary‐level data for acute pancreatitis was included from two independent cohorts. To make the UK Biobank summary statistics compatible with the GRCh38 reference assembly used by the FinnGen cohort, MungeSumstats tool^43^ was used to convert the UK Biobank data from the GRCh37 assembly to the GRCh38 assembly. We eliminated gene variants with low confidence and focused solely on those with a minor allele frequency greater than 0.01 for our meta‐analysis. A *z*‐score meta‐analysis of acute pancreatitis summary statistics was conducted between the FinnGen and the UK Biobank samples using METAL.^44^ The meta‐cohort for acute pancreatitis ultimately included a total of 6396 cases and 728,042 controls. After rigorous quality control, we narrowed down the variants from both cohorts to only those that met our standards. The resulting set of 14,410,399 variants was then used for the meta‐analysis in this study.

### 4.3 Mendelian randomization primary and sensitivity analyses

The effect of serum folate, homocysteine, and VB_12_ on acute pancreatitis outcomes was estimated using the random‐effects inverse variance weighted (IVW), MR‐Egger, and weighted median. The IVW method was applied to obtain the primary outcome. MR‐Egger and weighted median methods were performed to verify the causal effect. We harmonized SNPs to ensure that the effect estimates of each SNP on each trait and the risk of acute pancreatitis corresponded to the same allele. To assess the heterogeneity across each SNP, we employed Cochran's *Q* statistic and leave‐one‐out analysis. Additionally, we utilized the MR‐Egger intercept test and the MR‐PRESSO global test to detect potential horizontal pleiotropy. All these analyses were carried out using TwoSample MR (version 0.5.6)^45^ and MRPRESSO (version 1.0) in R (version 4.2.0).

### 4.4 Mice strains

Mice were group‐housed in individually ventilated plastic cages, maintained on a 14 h light and 10 h dark cycles, and provided with a standard chow diet (Chengdu Dashuo Experimental Animal Ltd.) as well as autoclaved water, both with ad libitum access. C57BL/6J wild‐type mice and the *CD320*‐ablation mice (C57BL/6J‐*CD320^em1C^*/Cya) were purchased from GemPharmatech and Cyagen Bioscience, respectively. For genotyping, genomic DNA obtained from the tails of 3‐week‐old offspring (*CD320* ^−/−^) mice and subjected to genotyping by polymerase chain reaction (PCR) and sequencing.

The total RNA was extracted from mouse pancreatic tissues with an RNA Extract Kit (TIANGEN, DP422). cDNA was generated using *Evo M‐NLV* RT‐PCR Kit (Accurate Biology, AG11601), and PCR analysis was performed. All primer sequences are listed in Table S3.

### 4.5 Induction of acute pancreatitis and treatment

The C57BL/6J wild‐type and *CD320* ^−/−^ mice, aged 6−8 weeks, were used and randomly divided into groups with each group containing 5−10 mice. Acute pancreatitis was induced by intraperitoneal injections of 20% L‐arginine solution (L‐Arg, Sigma‒Aldrich, A1513) at 3.3 g/kg per hour for 2 h. L‐Arg solution was prepared in saline (0.9% sodium chloride) and filter sterilized. Control mice were injected with sterile saline. For VB_12_ (Sigma‒Aldrich, 68‐19‐9) pretreatment, C57BL/6J wild‐type mice received four intraperitoneal injections of VB_12_ (500 µg/kg) 72, 48, and 24 h before L‐Arg injection. For VB_12_ treatment after L‐Arg‐induced pancreatitis, C57BL/6J wild‐type mice received three intraperitoneal injections of VB_12_ (500 µg/kg) 1, 3, and 5 h after L‐Arg injection. All mice were anesthetized with 1% isoflurane and euthanized 72 h after the administration of L‐Arg injection. For CER (TOCRIS, 6264) induced acute pancreatitis (CER‐AP) in wild‐type and *CD320* ^−/−^ mice, the CER‐AP group received seven intraperitoneal injections of 100 µg/kg CER hourly, with saline as controls. The CER + VB_12_, CER + GSH (Sigma‒Aldrich, G6013), and CER + ATP groups received three intraperitoneal injections of VB_12_ (500 µg/kg), GSH (500 mg/kg), and ATP (200 mg/kg, Sigma‒Aldrich, A6419‐1G), respectively, which were administered at 2, 4, and 6 h after the first injection of CER. All mice were anesthetized with 1% isoflurane and euthanized 12 h after the first CER injection. Blood serum and the pancreatic tissues were collected for further experiments.

### 4.6 Measurement of amylase and lipase in the serum

Serum amylase (C016) and lipase (A054) levels were assayed precisely following the manufacturer's instructions from Nanjing Jiancheng Bioengineering Institute. In brief, serum samples were collected by centrifugation, and diluted with saline for drawing the dose‒response curve. After incubated with substrate reaction solution, absorbance at 660 and 420 nm were measured for serum amylase and lipase, respectively.

### 4.7 Pancreatic trypsin activity

A fluorometric assay was performed to detect pancreatic trypsin activity as previously described.^46^ Briefly, pancreas tissues were homogenized in ice‐cold pH 6.5 buffer (5 mM MOP, 1 mM MgSO_4_, and 250 mM sucrose), centrifuged, and the supernatant was collected. The sample supernatant was detected with reaction buffer (50 mM Tris‒HCl, pH 8.0, 150 mM NaCl, 1 mM CaCl, 0.1 mg/mL bovine serum albumin [BSA]) containing Boc‐Gln‐Ala‐Arg‐MCA (Peptide, 3135‐v) at excitation wavelength of 380 nm and emission wavelength of 440 nm using a plate reader (CLARIOstar, BMG Labtech) for 10 min. A standard curve was obtained from purified human trypsin. The pancreatic protein concentration was detected using a BCA protein analysis (P0012, Beyotime).

### 4.8 Pancreatic MPO activity

Pancreatic MPO activity was measured using the substrate 3,3′,5,5′‐tetramethylbenzidine (TMB, Sigma‒Aldrich, 54827‐17‐7) as previously described.^46^ Briefly, the pancreas tissues were homogenized in 20 mM phosphate buffer (pH 7.4), centrifuged, and the supernatant was collected. The supernatant was mixed with a phosphate buffer (100 mM, pH 5.4) containing 0.5% HETAB and 20 mM TMB. The mixture was incubated at 37°C for 3 min, followed by the addition 0.01% H_2_O_2_, and further incubated for another 3 min. The difference in absorbance between 0 and 3 min at 655 nm was detected using a plate reader. A standard curve was obtained from purified human MPO.

### 4.9 Detection of VB_12_ in the serum and pancreatic tissues

The concentrations of VB_12_ in serum and pancreatic tissues were detected using an ELISA (Shanghai Enzyme‐Linked Biotechnology Co., Ltd., YJ057867). According to the manufacturer's instructions, standard solution and samples were added into the wells. Subsequently, the biotinylated VB_12_ detection antibody was added and the wells were incubated at 37°C for 1 h. After washing four times, and the wells were then sequentially incubated with streptavidin‒ Horseradish peroxidase (HRP) at 37°C for 40 min, washed four times, incubated with TMB substrate at 37°C for 30 min. A stop solution was added and detected immediately at 450 nm using a plate reader (SYNERGY H1, BioTek). The concentrations of VB_12_ were calculated based on standard curves. Additionally, the pancreatic tissue protein was determined by a BCA assay.

### 4.10 Measurement of ATP level in pancreatic tissues

For the detection of ATP levels, pancreatic tissues were collected and analyzed according to the Enhanced ATP Assay Kit protocol (Beyotime Biotechnology, S0027). In brief, pancreatic tissues were lysed using ATP detection lysis solution and centrifugated. The supernatant was collected and detected ATP content that calculated based on a standard ATP curve normalized to total protein according to the manufacturer's instruction.

### 4.11 Hematoxylin and eosin, immunohistochemistry staining, and immunofluorescence

For paraffin‐embedded sections, the fresh pancreatic specimens from mice were collected, fixed with 10% formalin for 72 h, and embedded in paraffin. Hematoxylin and eosin (H&E) and immunohistochemistry staining were performed as previously described.^47^ Sections (5 µm thick) were dewaxed, rehydrated, and stained with H&E. For the immunohistochemistry staining, tissue antigen retrieval was conducted by boiling in 10 mM (pH 6.0) citrate buffer (Sigma) for 45 min. Sections were allowed to cool to room temperature before treatment of 3% H_2_O_2_ and then hybridized with primary antibodies of CD45R (1:100, Santa Cruz, c‐19597), cytokeratin 19 (CK‐19) (1:100, Santa Cruz, sc‐376126), CD11b (1:100, eBioscience, M1/70), and CD3 (1:100, Beyotime Biotechnology, AF1480) overnight at 4°C. Sections were then stained with secondary antibodies (ZSGB‐Bio) for 1 h at room temperature. Negative and positive controls were applied in each staining run. CK‐19‐positive metaplasia and the infiltration of CD45R^+^, CD11b^+^, and CD3^+^ cells were manually quantified from five random and non‐overlapping fields and averaged for each mouse as described previously.^48^

For frozen sections, the fresh pancreatic specimens from mice were collected, fixed with 4% polyformaldehyde (PFA) at RT for 1 h, dehydrated with 30% sucrose overnight, and embedded in optimal cutting temperature compound (Sakura, 4583). For immunofluorescence, slides (5 µm thick) were fixed with 4% PFA, washed with phosphate‐buffered saline (PBS), and blocking for 1 h with 4% BSA containing 0.5% Triton X‐100. Primary antibodies were diluted in 4% BSA containing 0.5% Triton X‐100 and incubated overnight at 4°C or 1 h at RT. Secondary antibodies were diluted in PBS with 0.1% Tween‐20 and incubated for 1 h at RT. Slides were mounted in Antifade Mounting Medium with 4'6‐diamidino‐2‐phenylindole (Beyotime Biotechnology, P0131).

### 4.12 Flow cytometry

The fresh pancreatic tissue was digested with collagenase to form a single‐cell suspension and then cryopreserved in liquid nitrogen. The cells were resuscitated at 37°C and washed with Dulbecco's phosphate‐buffered saline (DPBS). Subsequently, the cells were labeled with Fixable viability stain 700 (FVS700) and stained with fluorescent antibodies (BioLegend, CD3‐BV421, CD4‐FITC, CD8‐APC, CD45‐BV510, and CD25‐PE). After washing with DPBS again, the cells were labeled with Foxp3 (BioLegend, PerCP‐Cy5.5) using the Foxp3 staining kit (eBioscience, 00‐5523‐00). Finally, the cells were analyzed by flow cytometer (BD Celesta).

### 4.13 Histological scoring for acute pancreatitis

Histopathology scores for acute pancreatitis were evaluated basic on edema, inflammatory cell infiltration, and necrosis as previously reported.^49^, ^50^ In each experimental group, two separate and blinded evaluators assessed 10 randomly selected areas on each pancreatic slide, utilizing a 200× magnification. The degree of pancreatic damage was quantified by evaluating the presence and extent of edema, inflammatory cell infiltration, necrosis, and then calculating a comprehensive histopathology score that is the aggregate of individual scores for these features.^49^

### 4.14 Data and statistical analysis

The methodology for data collection and statistical analysis in our study adhered to the guidelines established in pharmacology for experimental design and analysis.^51^ All experiments were repeated at least three times, and all data are expressed as mean ± SD. We compared the results of the treatment groups and controls in mice using one‐way analysis of variance (ANOVA) and Tukey's multiple‐comparison posttest or an unpaired *t*‐test. Differences between groups were significant at a *p*‐value of <0.05. Statistical analyses were performed using GraphPad Prism 9.0 (GraphPad Software, Inc.).

## AUTHOR CONTRIBUTIONS

*Data validation, analysis, research design, and execution*: Yulin Chen, Xue Li, and Ran Lu. *Performed experiments*: Junman Ye, Li Li, Yinchun Lv, Qiaorong Huang, Jin Zhao, and Yongzi Wu. *Data collection, data validation, and analysis*: Junman Ye, Wentong Meng, Feiwu Long, Qing Xia, and Wei Huang. *Research design, conceptualization, supervision, data validation, analysis, and interpretation*: Chuanwen Fan and Xianming Mo. *Conceived and designed the study and oversaw all analyses*: Yulin Chen, Xue Li, Ran Lu, and Chuanwen Fan. All authors read and approved the final version of this manuscript.

## CONFLICT OF INTEREST STATEMENT

The authors declare they have no conflicts of interest.

## ETHICS STATEMENT

The publicly available summary statistics for acute pancreatitis were obtained from the UK Biobank study (Neale Lab) and the FinnGen consortium, and no original data were involved in the current MR study. The indicated consortia described ethical approval and informed consent from every individual for the corresponding studies included in their research. All animal experimental protocols were approved by the Ethical Committee of West China Hospital Sichuan University, Chengdu, China (20230106003).

## Supporting information

Supporting Information

## Data Availability

All summary‐level data for analysis in this study are available on the website https://www.decode.com/summarydata/ for GWAS of serum folate, homocysteine, and VB_12_; https://r7.finngen.fi/ for the FinnGen released 7 data; and https://yanglab.westlake.edu.cn/data/ukb_fastgwa/imp_binary/ for the UK Biobank cohort data. All data are available within the article, Supporting Information, or available from the corresponding author upon reasonable request.
