# Supplementary material for: Vitamin B12 protects necrosis of acinar cells in pancreatic tissues with acute pancreatitis
Source: MedComm (2020). 2024 Oct 15;5(11):e686. doi: 10.1002/mco2.686 (PMC11480517; doi:10.1002/mco2.686)
Supplement: Supplementary file 1 — Supporting Information [file MCO2-5-e686-s001.docx]

**SUPPLEMENTAL MATERIAL**

**Vitamin B_12_ protects necrosis of acinar cells in pancreatic tissues with acute pancreatitis**

Yulin Chen^1,8^, Xue Li^1,8^, Ran Lu^1,2,3,8^, Yinchun Lv^1^, Yongzi Wu^1^, Junman Ye^1^, Jin Zhao^1^, Li Li^6^, Qiaorong Huang^1^, Wentong Meng^1^, Feiwu Long^4^, Wei Huang^1^, Qing Xia^1^, Jianbo Yu^7^, Chuanwen Fan^4,5*^, Xianming Mo^1*^.

^1^ West China Center of Excellence for Pancreatitis, Institute of Integrated Traditional Chinese and Western Medicine, Laboratory of Stem Cell Biology, State Key Laboratory of biotherapy, West China Hospital, Sichuan University, Chengdu, China.

^2^ Department of Occupational and Environmental Health, West China School of Public Health and West China Fourth Hospital, Sichuan University, Chengdu, China.

^3^ West China-PUMC C. C. Chen Institute of Health, West China School of Public Health, and West China Fourth Hospital, Sichuan University, Chengdu, China.

^4^ Department of Gastrointestinal, Bariatric, and Metabolic Surgery, Research Center for Nutrition, Metabolism & Food Safety, West China-PUMC C.C. Chen Institute of Health, West China School of Public Health and West China Fourth Hospital, Sichuan University, Chengdu, China.

^5^ Department of Oncology and Department of Biomedical and Clinical Sciences, Linköping University, Linköping, Sweden.

^6^ School of Basic Medicine, Southwest Medical University, Luzhou, China.

^7^ Longgang Central Hospital, Shenzhen, China

^8^ These authors contributed equally.

^*^Correspondence: Chuanwen Fan: [chuanwen.fan@liu.se](mailto:chuanwen.fan@liu.se)

Xianming Mo: [xmingmo@scu.edu.cn](mailto:xmingmo@scu.edu.cn)

**This file includes:**

Tables. S1 to S3

Figures. S1 to S11

**Table S1. The summary of GWAS data and consortia in the present study**

| **Exposure/outcomes** | **No. of cases** | **No. of controls** | **Sample size** | **Number of SNPs** | **PubMed ID or web-link** | **Adjustments** |
| --- | --- | --- | --- | --- | --- | --- |
| Homocysteine | - | - | 44,147 | 14 | 23824729 | Age and sex, in the Rotterdam Study cohorts |
| VB_12_ | - | - | 45,576 | 14 | 23754956 | Sex, year of birth and age at measurement |
| Folate | - | - | 374,65 | 3 | 23754956 | Sex, year of birth and age at measurement |
| Acute pancreatitis | 4,648 | 273,442 | - | - | FinnGen consortium  (https://www.finngen.fi/fi) | Sex, age,10 principal components and genotyping batch |
| Acute pancreatitis | 1,748 | 454,600 | - | - | UK Biobank  (http://www.nealelab.is/ukbiobank) | Age, sex and up to 20 genetic principal components |

SNP: single-nucleotide polymorphisms; VB_12_, vitamin B_12_; PubMed ID indicates PubMed identifier.

**Table S2. The characteristic of instrumental variables in the exposure and outcome.**

|  | | | **Exposure** | | | |  | **FinnGen** | | |  | **UK Biobank** | | |  | **AP-meta** | | |  |
| --- | --- | --- | --- | --- | --- | --- | --- | --- | --- | --- | --- | --- | --- | --- | --- | --- | --- | --- | --- |
| **SNP** | **EA** | **OA** | **β** | **SE** | ***P*** | ***F*** |  | **β** | **SE** | ***P*** |  | **β** | **SE** | ***P*** |  | **β** | **SE** | ***P*** | **Nearby gene** |
| **Folate** | | | | | | | | | | | | | | | | | | | |
| rs652197 | C | T | 0.07 | 0.01 | 1.40E-12 | 362 |  | 0.01 | 0.03 | 0.61 |  | 0.05 | 0.05 | 0.36 |  | 0.02 | 0.02 | 0.39 | FOLR3 |
| rs1801133 | G | A | 0.1 | 0.01 | 9.50E-53 | 1056 |  | 0 | 0.02 | 0.88 |  | -0.02 | 0.04 | 0.66 |  | 0 | 0.02 | 0.89 | MTHFR |
| rs17421511 | G | A | 0.02 | 0.83 | 1.80E-15 | 554 |  | 0.02 | 0.03 | 0.49 |  | 0 | 0.05 | 0.96 |  | 0.01 | 0.02 | 0.58 | MTHFR |
| **Homocysteine** | | | | | | | | | | | | | | | | | | | |
| rs9369898 | A | G | 0.05 | 0.01 | 2.20E-10 | 646 |  | -0 | 0.02 | 0.28 |  | 0.01 | 0.04 | 0.76 |  | -0.01 | 0.02 | 0.46 | MUT |
| rs838133 | A | G | 0.04 | 0.01 | 7.50E-09 | 633 |  | 0.06 | 0.02 | 0 |  | NA | NA | NA |  | -0.06 | 0.02 | 0.01 | FUT2 |
| rs7130284 | C | T | 0.12 | 0.01 | 1.90E-20 | 262 |  | NA | NA | NA |  | -0.03 | 0.06 | 0.61 |  | 0.01 | 0.03 | 0.71 | NOX4 |
| rs548987 | C | G | 0.06 | 0.01 | 1.10E-08 | 287 |  | -0 | 0.04 | 0.7 |  | 0.04 | 0.05 | 0.39 |  | -0.02 | 0.03 | 0.41 | SLC17A3 |
| rs4660306 | T | C | 0.04 | 0.01 | 2.30E-09 | 578 |  | 0 | 0.02 | 0.93 |  | -0.08 | 0.04 | 0.03 |  | 0.02 | 0.02 | 0.22 | MMACHC |
| rs42648 | G | A | 0.04 | 0.01 | 2.00E-08 | 569 |  | 0 | 0.02 | 0.9 |  | -0.05 | 0.03 | 0.13 |  | 0.01 | 0.02 | 0.48 | GTPB10 |
| rs234709 | C | T | 0.07 | 0.01 | 3.90E-24 | 1096 |  | NA | NA | NA |  | 0.02 | 0.03 | 0.49 |  | -0.02 | 0.03 | 0.5 | CBS |
| rs2275565 | G | T | 0.05 | 0.01 | 2.00E-10 | 422 |  | 0.04 | 0.03 | 0.16 |  | 0.02 | 0.04 | 0.58 |  | 0.02 | 0.02 | 0.38 | MTR |
| rs2251468 | C | A | 0.05 | 0.01 | 1.30E-12 | 708 |  | 0 | 0.02 | 0.94 |  | -0.09 | 0.04 | 0.01 |  | NA | NA | NA | HNF1A |
| rs1801222 | A | G | 0.05 | 0.01 | 8.40E-10 | 615 |  | 0.02 | 0.02 | 0.4 |  | -0.04 | 0.04 | 0.22 |  | 0 | 0.02 | 0.97 | CUBN |
| rs1801133 | A | G | 0.16 | 0.01 | 4.30E-104 | 2236 |  | 0 | 0.02 | 0.88 |  | -0.02 | 0.04 | 0.66 |  | 0 | 0.02 | 0.89 | MTHFR |
| rs154657 | A | G | 0.1 | 0.01 | 1.70E-43 | 1484 |  | 0 | 0.02 | 0.86 |  | 0.05 | 0.03 | 0.13 |  | -0.01 | 0.02 | 0.51 | DPEP1 |
| rs12780845 | A | G | 0.05 | 0.01 | 7.80E-10 | 570 |  | 0.04 | 0.02 | 0.09 |  | 0.06 | 0.04 | 0.1 |  | -0.01 | 0.02 | 0.65 | CUBN |
| rs1047891 | A | C | 0.09 | 0.01 | 4.60E-27 | 1022 |  | -0 | 0.02 | 0.09 |  | -0.04 | 0.04 | 0.3 |  | -0.02 | 0.02 | 0.38 | CPS1 |
| **VB_12_** | | | | | | | | | | | | | | | | | | | |
| rs7788053 | A | G | 0.05 | 0.01 | 1.70E-10 | 532 |  | 0.01 | 0.03 | 0.86 |  | NA | NA | NA |  | NA | NA | NA | FUT6 |
| rs602662 | A | G | 0.16 | 0.01 | 2.40E-139 | 2469 |  | -0 | 0.02 | 0.25 |  | -0.06 | 0.03 | 0.08 |  | -0.03 | 0.02 | 0.06 | FUT2 |
| rs56077122 | A | C | 0.09 | 0.01 | 4.80E-21 | 945 |  | 0.04 | 0.02 | 0.1 |  | 0.07 | 0.04 | 0.07 |  | 0.01 | 0.02 | 0.74 | CUBN/TRDMT1 |
| rs41281112 | C | T | 0.17 | 0.02 | 8.90E-35 | 173 |  | 0.1 | 0.06 | 0.1 |  | 0.22 | 0.11 | 0.04 |  | 0.02 | 0.05 | 0.71 | CLYBL |
| rs3742801 | T | C | 0.05 | 0.01 | 1.70E-13 | 444 |  | 0.01 | 0.02 | 0.67 |  | -0.03 | 0.04 | 0.4 |  | 0.02 | 0.02 | 0.42 | ABCD4 |
| rs34528912 | T | C | 0.17 | 0.02 | 2.10E-15 | 133 |  | 0.02 | 0.05 | 0.7 |  | 0.13 | 0.09 | 0.14 |  | -0.01 | 0.04 | 0.72 | TCN1 |
| rs34324219 | C | A | 0.21 | 0.01 | 1.10E-111 | 1394 |  | 0.04 | 0.03 | 0.29 |  | 0 | 0.05 | 0.93 |  | 0.03 | 0.03 | 0.36 | TCN1 |
| rs2336573 | T | C | 0.32 | 0.01 | 8.40E-59 | 575 |  | -0 | 0.07 | 0.87 |  | -0.02 | 0.09 | 0.87 |  | 0 | 0.05 | 0.98 | CD320 |
| rs2270655 | G | C | 0.07 | 0.02 | 2.20E-13 | 88 |  | -0 | 0.05 | 0.43 |  | -0.02 | 0.08 | 0.76 |  | -0.02 | 0.04 | 0.61 | MMAA |
| rs1801222 | G | A | 0.11 | 0.01 | 3.30E-75 | 1683 |  | 0.02 | 0.02 | 0.4 |  | -0.04 | 0.04 | 0.22 |  | 0 | 0.02 | 0.97 | CUBN |
| rs12272669 | A | G | 0.51 | 0.01 | 3.00E-09 | 310 |  | NA | NA | NA |  | 0.07 | 0.07 | 0.27 |  | -0.07 | 0.07 | 0.27 | MMACHC |
| rs117456053 | G | A | 0.16 | 0.03 | 1.90E-09 | 52 |  | 0 | 0.06 | 0.98 |  | -0.12 | 0.16 | 0.44 |  | 0.02 | 0.06 | 0.79 | TCN1 |
| rs1141321 | C | T | 0.06 | 0.01 | 3.60E-26 | 884 |  | -0 | 0.02 | 0.28 |  | 0.01 | 0.04 | 0.81 |  | NA | NA | NA | MUT |
| rs1131603 | C | T | 0.19 | 0.02 | 4.90E-49 | 271 |  | -0.1 | 0.04 | 0.14 |  | 0.2 | 0.08 | 0.02 |  | 0.08 | 0.03 | 0.02 | TCN2 |

EA, effect allele; OA, other allele; VB_12_, vitamin B_12_; NA, not available; SE, standard error; SNP, single-nucleotide polymorphisms; *P*, *P*-value; AP, acute pancreatitis. AP-meta is combined of data of the FinnGen consortium and the UK Biobank cohort.­­

**Table S3. The sequence of primer for genotyping and RT-PCR.**

| **Primer** | **Sequence** |
| --- | --- |
| *CD320*-F1 | GACCATTGGGCAGTGATATTTGC |
| *CD320*-R1 | AACAGGGGATAGCCAAGTTCAAATC |
| *CD320*-R2 | CCATCCAACAGCTAGCCGAAC |
| *CD320*-F3(RT-PCR) | CCTTATTCCTGCGCTGTCC |
| *CD320*-R3(RT-PCR) | CAATCCTACAGTCTTCCTCGT |
| *CD320*-F4(RT-PCR) | ACTACAAGGATTTCTACGACCA |
| *CD320*-R4(RT-PCR) | TGTTTCACGGCTCCTTTGTCC |
| *GAPDH*-F1 | GTGAACCACGAGAAATATGACAAC |
| *GAPDH*-R1 | AGTGATGGCATGGACTGTG |

NOTE. For genotyping, Wild-type allele, one band with 652 bp. Heterozygotes, two bands with 652 bp and 952 bp, and Homozygotes, one band with 952 bp.


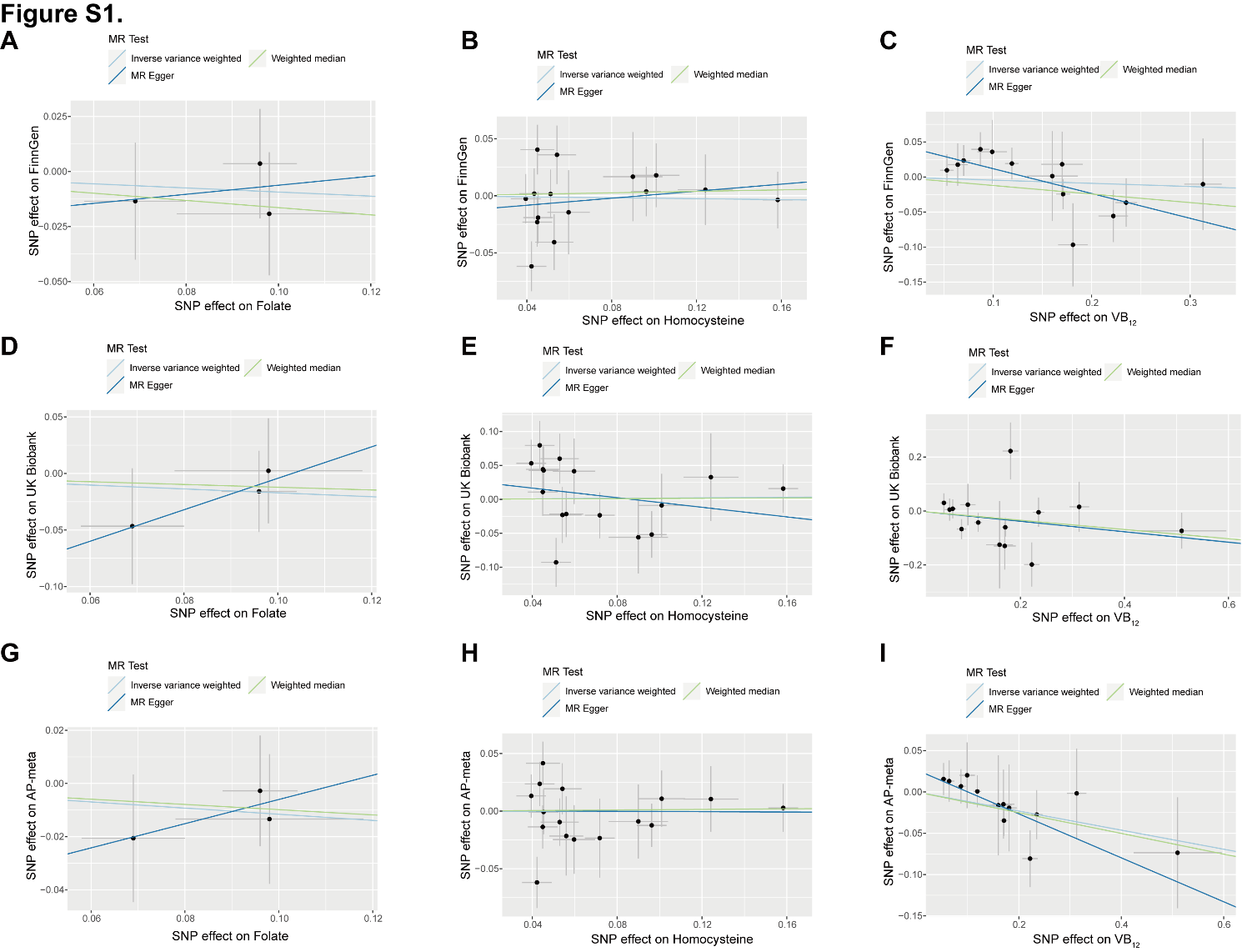


**Figure S1. The scatter plots for MR analyses in different cohorts.**

(A) Folate, (B) homocysteine, and (C) VB_12_ in the FinnGen consortium. (D) Folate, (e) homocysteine, and (F) VB_12_ in the UK Biobank cohort. (G) Folate, (H) homocysteine, and (I) VB_12_ in the AP-meta. AP, acute pancreatitis.


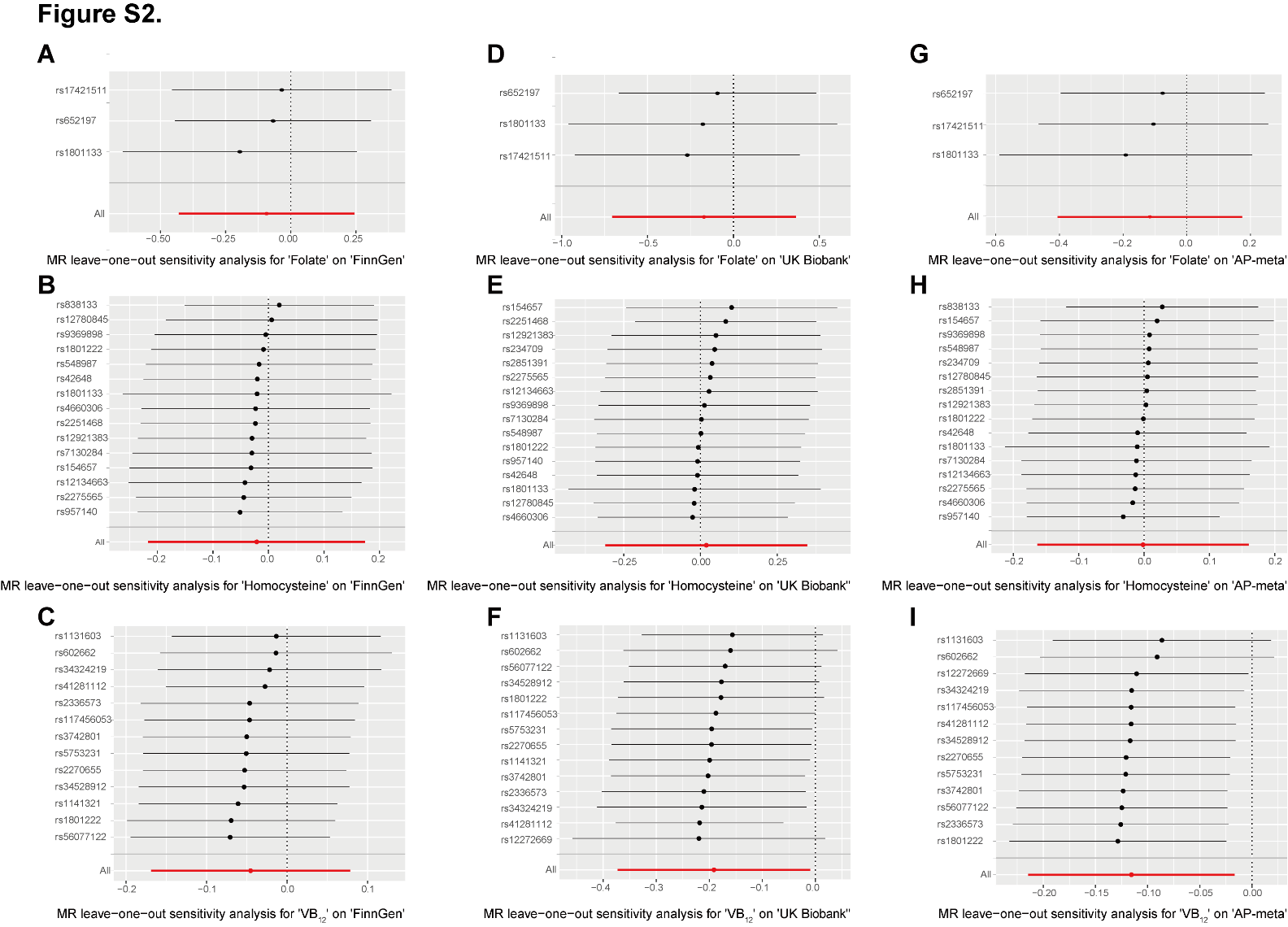


**Figure S2. The representative images of leave-one-out analyses for MR analyses in different cohorts.**

(A) Folate, (B) homocysteine, and (C) VB_12_ in the FinnGen consortium. (D) Folate, (e) homocysteine, and (F) VB_12_ in the UK Biobank cohort. (G) Folate, (H) homocysteine, and (I) VB_12_ in the AP-meta. AP, acute pancreatitis.

**
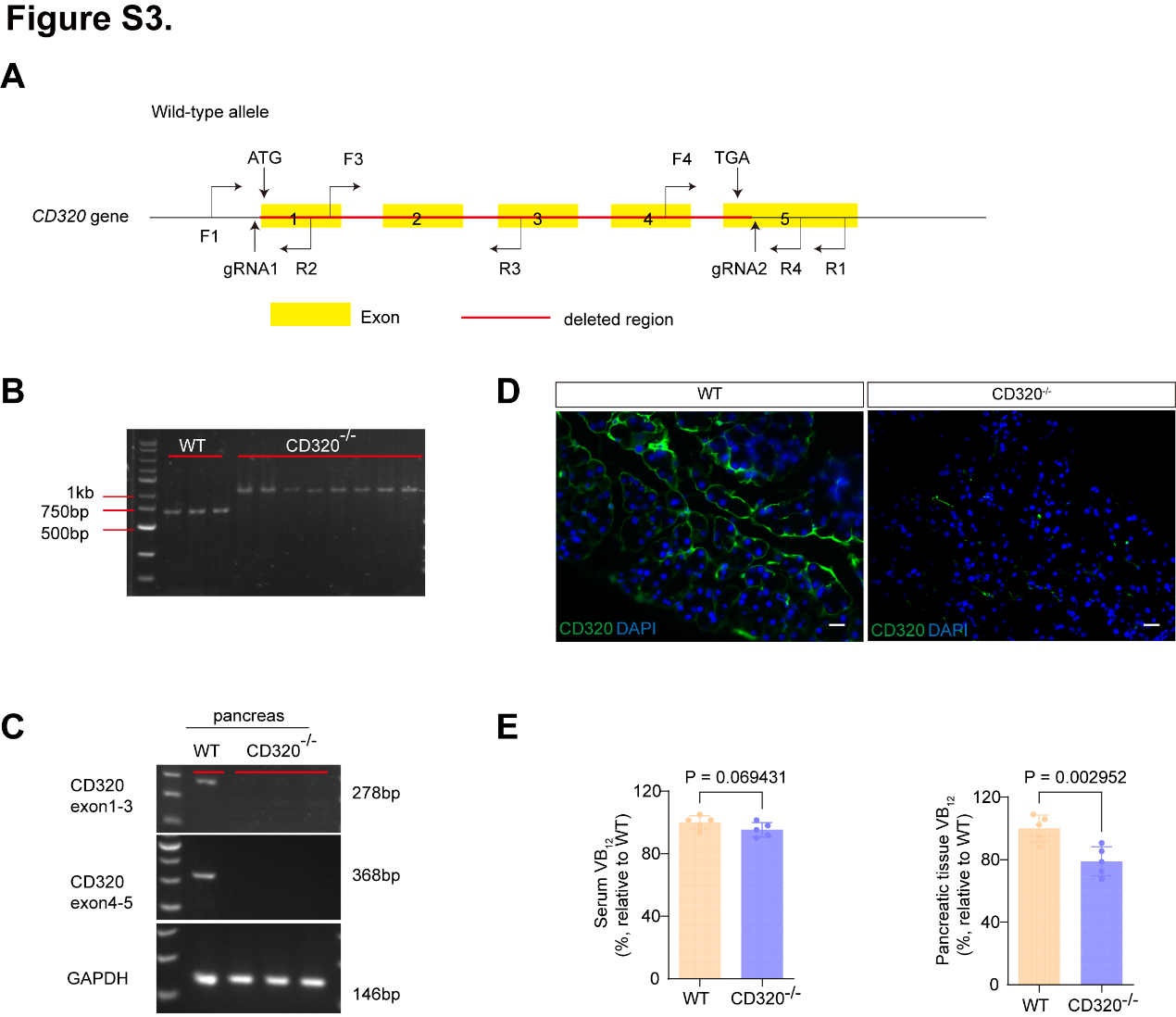
**

**Figure S3. Identifying the *CD320*-ablation mice.**

(A) The diagram of strategy for identifying the *CD320*^-/-^ mice. (B) Representative images of genotyping of wild-type and *CD320*^-/-^ mice using gDNA from tail clips. Two different sets of primers (F1+R1 and F1+R2) amplifying *CD320* were used. Wild-type allele, one band with 652 bp. Heterozygotes, two bands with 652 bp and 952 bp, and Homozygotes, one band with 952 bp. (C) Representative images of RT-PCR of wild-type and *CD320*^-/-^ mice using cDNA from pancreatic tissue in wild-type and *CD320*^-/-^ mice, the primers (F3+R3 and F4+R4) amplifying the exon of *CD320* were used, and *GAPDH* was amplified as a control. (D) Representative immunofluorescence images for *CD320* of pancreatic tissue in wild-type and *CD320*^-/-^ mice. (E) The level of VB_12_ in serum and pancreatic tissue, data were relative to wild-type mice group and presented as means ± SD with *student*’s t-test.


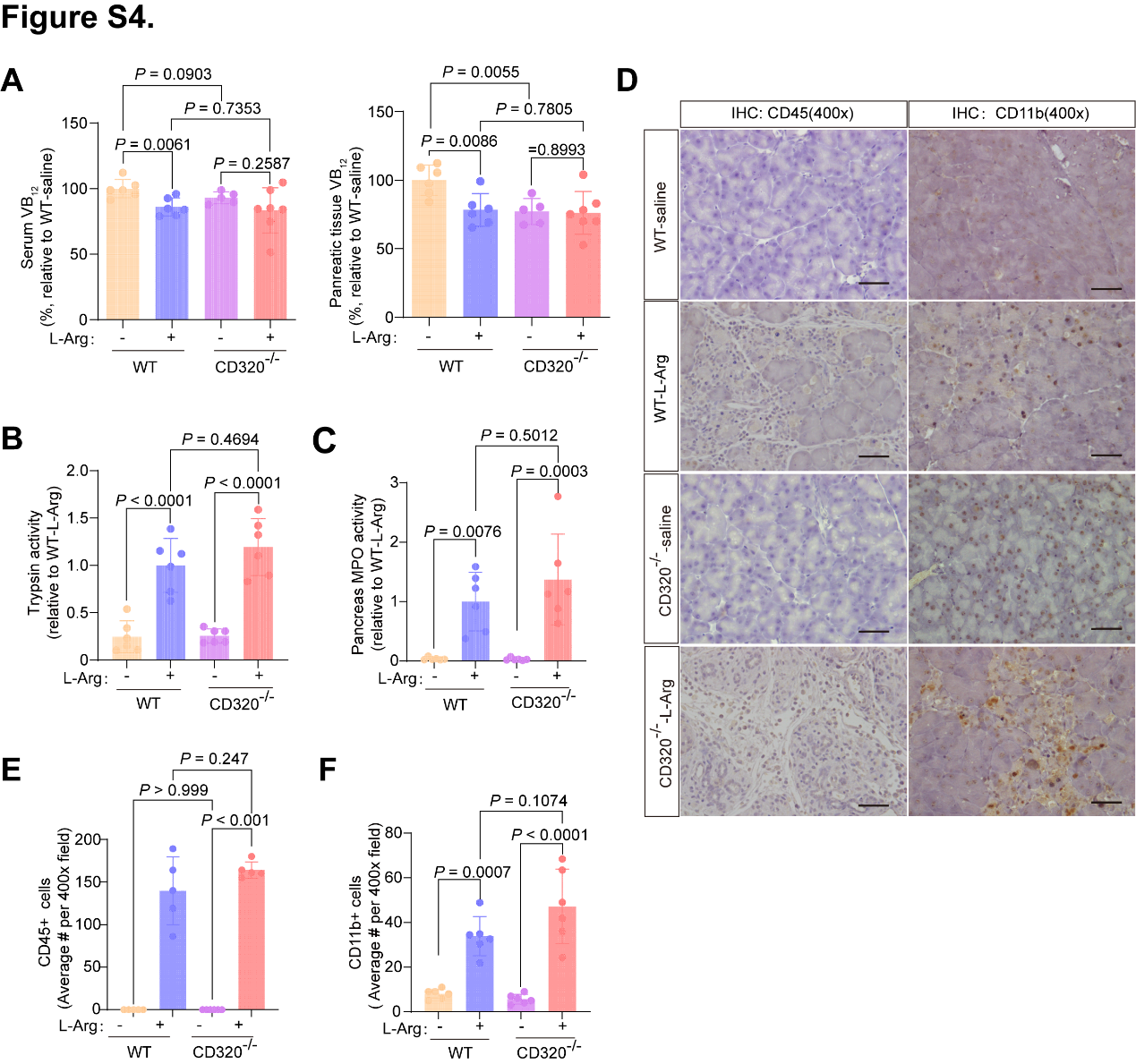


**Figure S4. Acute pancreatitis induced by L-Arg is aggravated in *CD320*-ablation mice.**

(A) The level of VB_12_ in serum and pancreatic tissue at 72 hours after the final administration of L-Arg. (B, C) Representative of activity of trypsin and MPO in pancreatic tissue at 72 hours after the final administration of L-Arg, respectively. (D) Representative immunohistochemical images for CD45 and CD11b of mouse pancreatic tissue at 72 hours after the final administration of L-Arg (Scale bar, 50 μm). (E, F). Quantification of immunohistochemical analysis for CD45 and CD11b of mouse pancreatic tissue at 72 hours after the final administration of L-Arg, respectively. Data were all presented as means ± SD from individual mice with one-way analysis of variance (ANOVA) and Tukey's multiple-comparison posttest or an unpaired *t*-test.


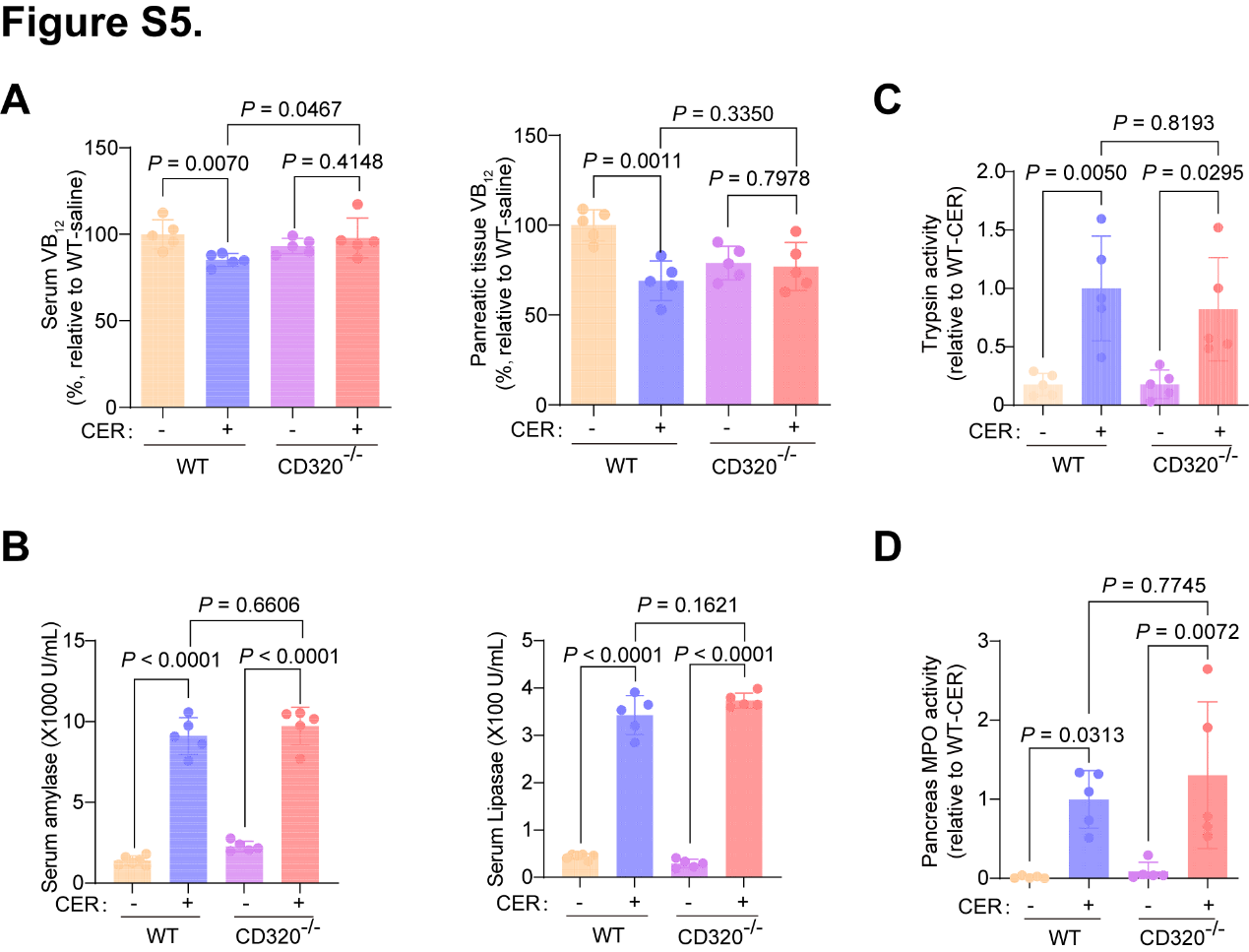


**Figure S5.** **Acute pancreatitis induced by CER is aggravated in *CD320*-ablation mice.**

(A) The level of VB_12_ in serum and pancreatic tissue at 12 hours after the first administration of CER. (B) The levels of serum amylase and lipase were measured at 12 hours after the first administration of CER. (C, D) Representative of activity of trypsin and MPO in pancreatic tissue at 12 hours after the first administration of CER, respectively. Data were from 5 mice per group and presented as mean±SD from individual mice with one-way analysis of variance (ANOVA) and Tukey's multiple-comparison posttest or an unpaired *t*-test.


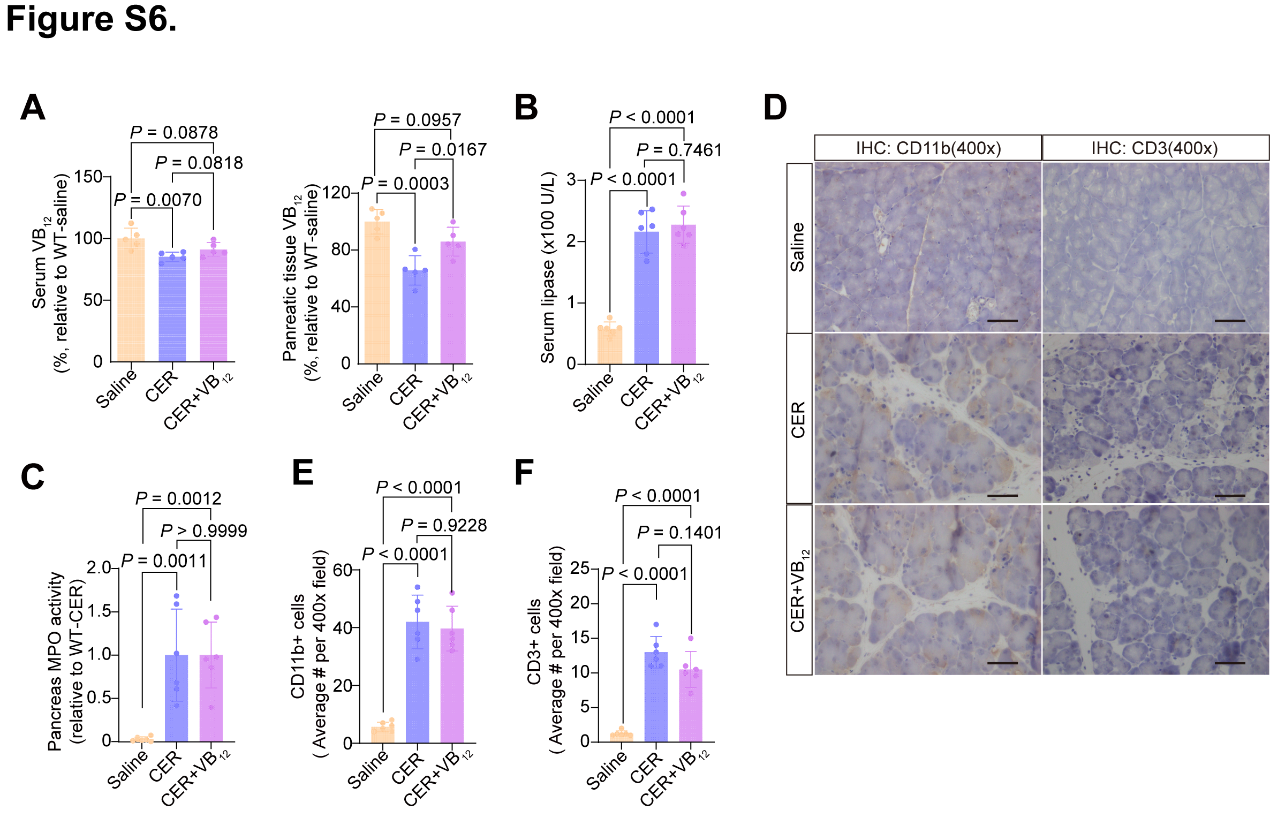


**Figure S6. Vitamin B_12_ displays therapeutic effects on acute pancreatitis induced by CER in mouse models.**

(A) The level of VB_12_ in serum and pancreatic tissue at 12 hours after the first CER treatment. (B) Serum lipase levels were measured at 12 hours after the first administration of CER treatment. (C) Representative of activity of MPO in pancreatic tissue at 12 hours after the first administration of CER. (D) Representative immunohistochemical images for CD11b+ and CD3+ cell of mouse pancreatic tissue at 12 hours after the first administration of CER (Scale bar, 50 mm). (E, F) Quantification of immunohistochemical analysis for CD11b+ and CD3+ cell of mouse pancreatic tissue at 12 hours after the first administration of CER, respectively. Data was from 5-6 mice per group and presented as mean±SD from individual mice with one-way analysis of variance (ANOVA) and Tukey's multiple-comparison posttest or an unpaired *t*-test.


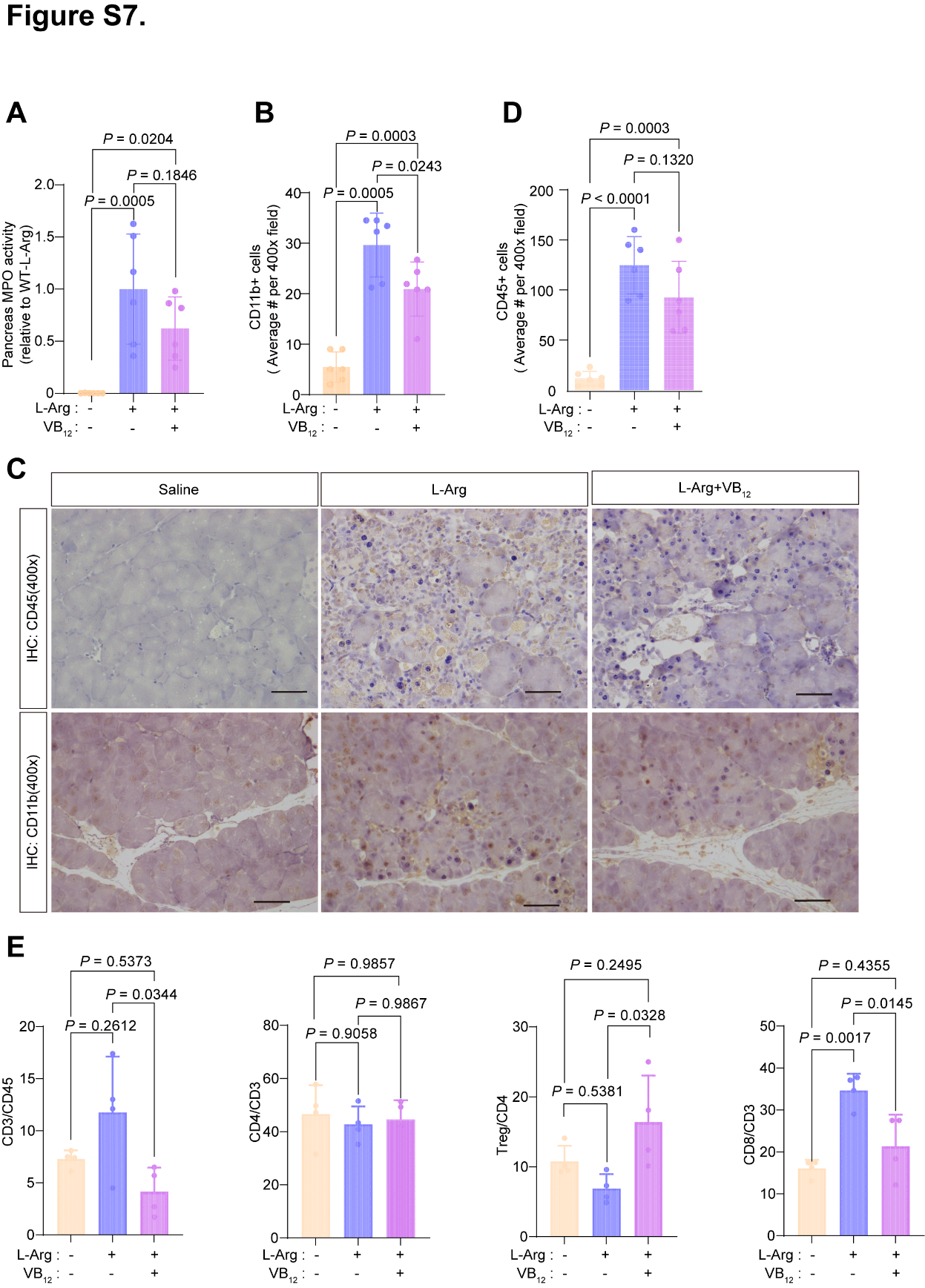


**Figure S7. Vitamin B_12_ displays therapeutic effects on acute pancreatitis induced by L-Arg in mouse models.**

(A, B) Representative of activity of trypsin and MPO in pancreatic tissue at 72 hours after the final administration of L-Arg, respectively. (C) Representative immunohistochemical images for CD45+ and CD11b+ cell of mouse pancreatic tissue at 72 hours after the final administration of L-Arg (Scale bar, 50 μm). (D, E). Quantification of immunohistochemical analysis for CD45+ and CD11b+ cell of mouse pancreatic tissue at 72 hours after the final administration of L-Arg, respectively. (E) Detect the immune cells in mouse pancreatic tissue at 72 hours after the final administration of L-Arg. Data are all presented as mean±SD from individual mice with one-way analysis of variance (ANOVA) and Tukey's multiple-comparison posttest.


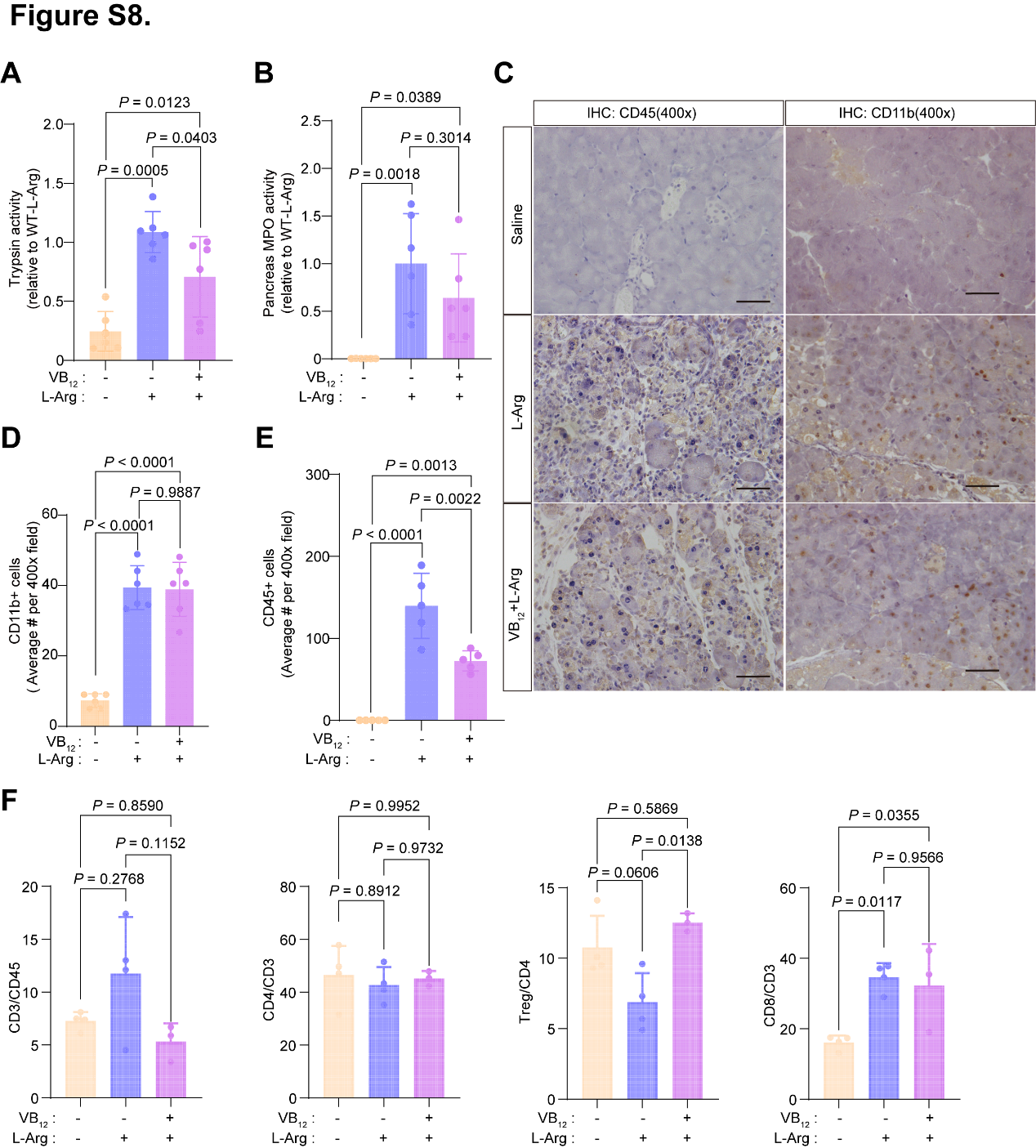


**Figure S8. Vitamin B_12_ displays preventative effects on acute pancreatitis induced by L-Arg in mouse models.**

(A, B) Representative of activity of trypsin and MPO in pancreatic tissue at 72 hours after the final administration of L-Arg, respectively. (C) Representative immunohistochemical images for CD11b+ and CD45+ cell of mouse pancreatic tissue at 72 hours after the final administration of L-Arg (Scale bar, 50 μm). (D, E). Quantification of immunohistochemical analysis for CD11b+ and CD45+ cell of mouse pancreatic tissue at 72 hours after the final administration of L-Arg, respectively. (F) Detect the immune cells in mouse pancreatic tissue at 72 hours after the final administration of L-Arg. Data are all presented as mean±SD from individual mice with one-way analysis of variance (ANOVA) and Tukey's multiple-comparison posttest.


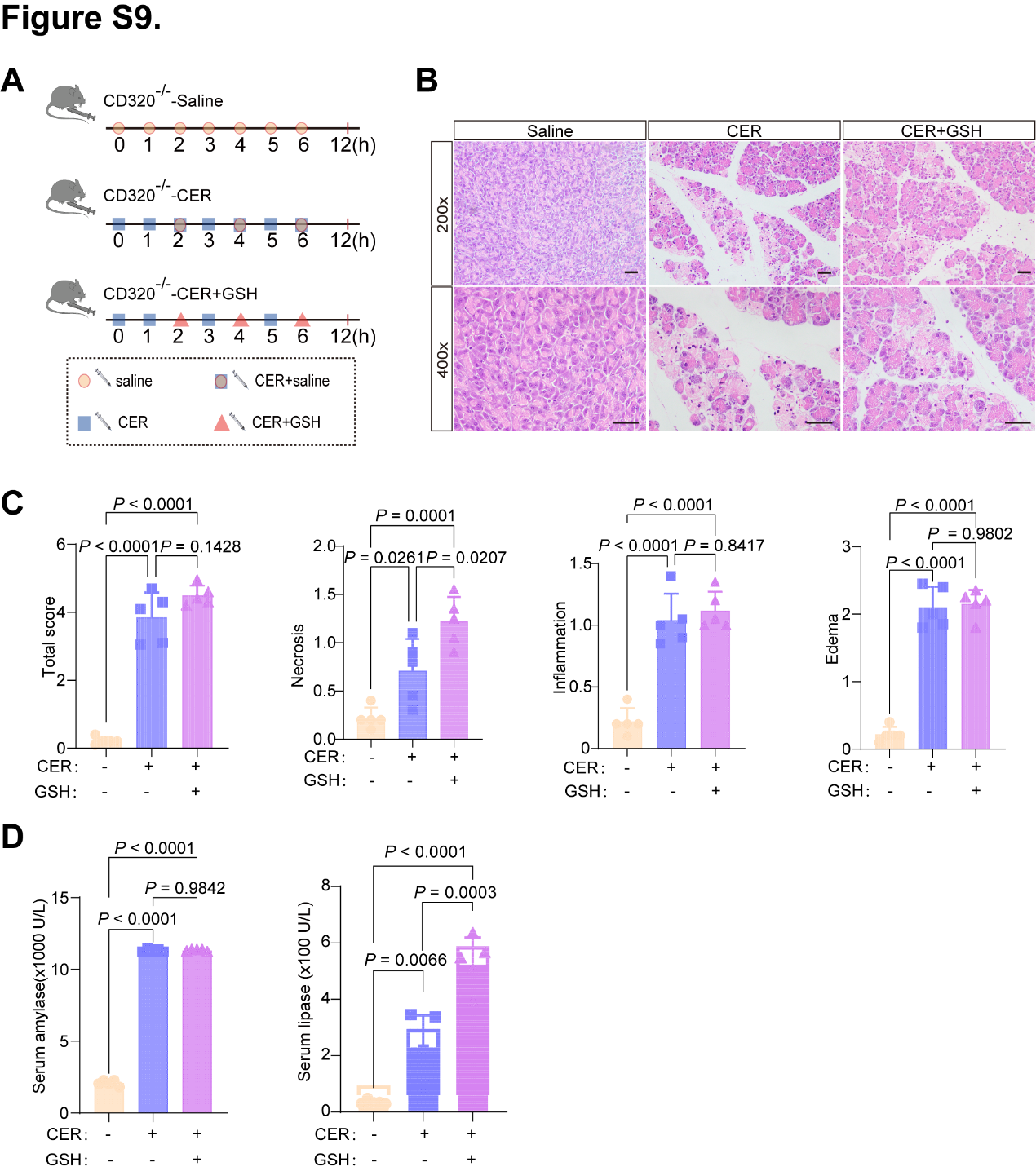


**Figure S9. GSH displays not therapeutic effects on acute pancreatitis induced by CER in mouse models.**

(A) The flow charts of experiment. (B) The representative histological were obtained 12 hours after the first administration of CER (Scale bar, 50 μm). (C) The histological evaluation of pancreatic edema, inflammatory infiltration, necrosis, and the overall sum of these features. (D) Serum amylase and lipase levels were measured at 12 hours after the first administration of CER treatment.


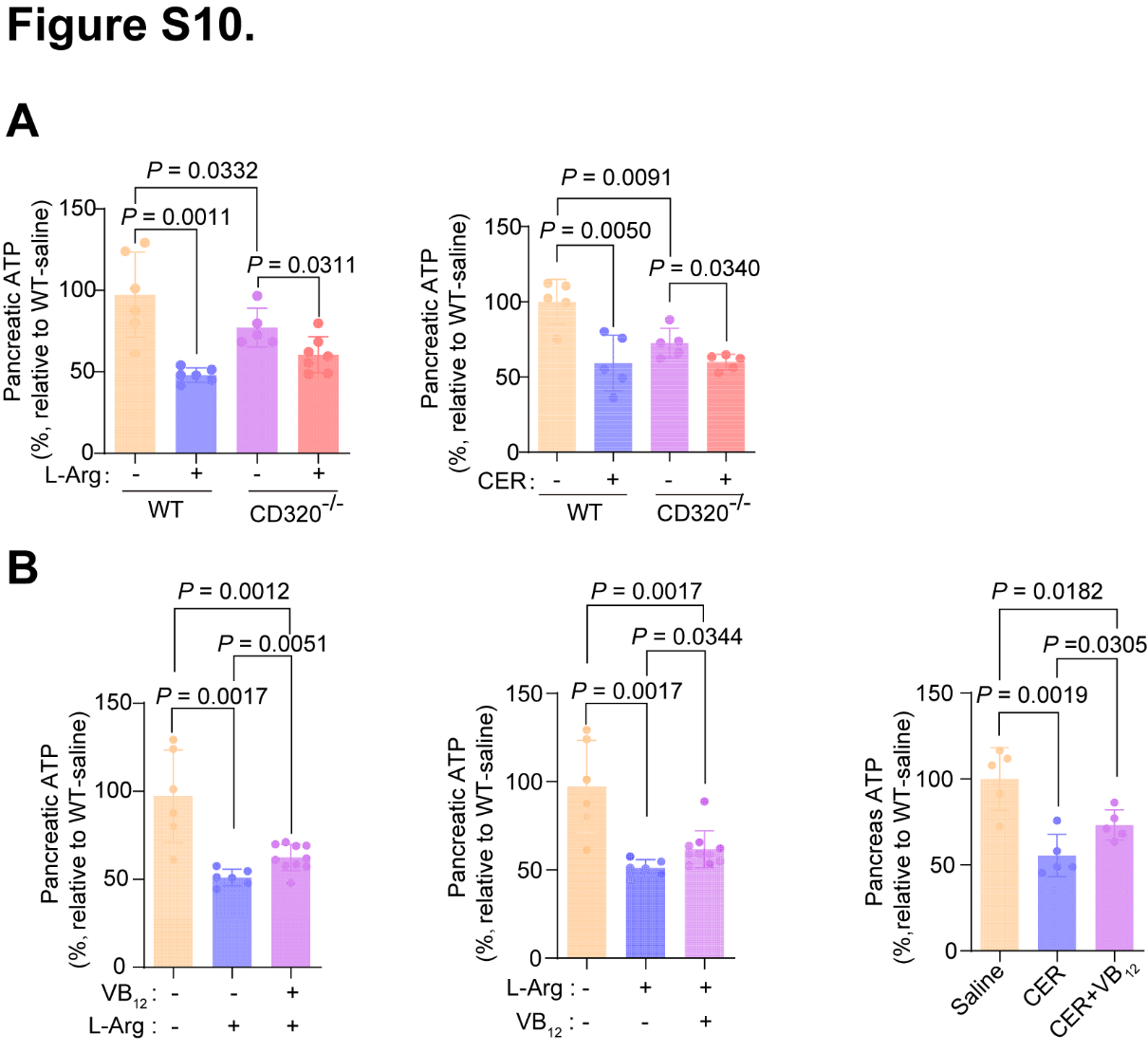


**Figure S10. The level of pancreatic ATP was detected after pretreatment and treatment with vitamin B_12_ in pancreatitis mouse models.**

(A) The levels of ATP in pancreatic tissue in pancreatitis *CD320*-ablation mouse model. (B) The levels of ATP in pancreatic tissue in preventive and therapeutic mouse model. Data were from 5-10 mice per group and presented as mean±SD from individual mice with one-way analysis of variance (ANOVA) and Tukey's multiple-comparison posttest.


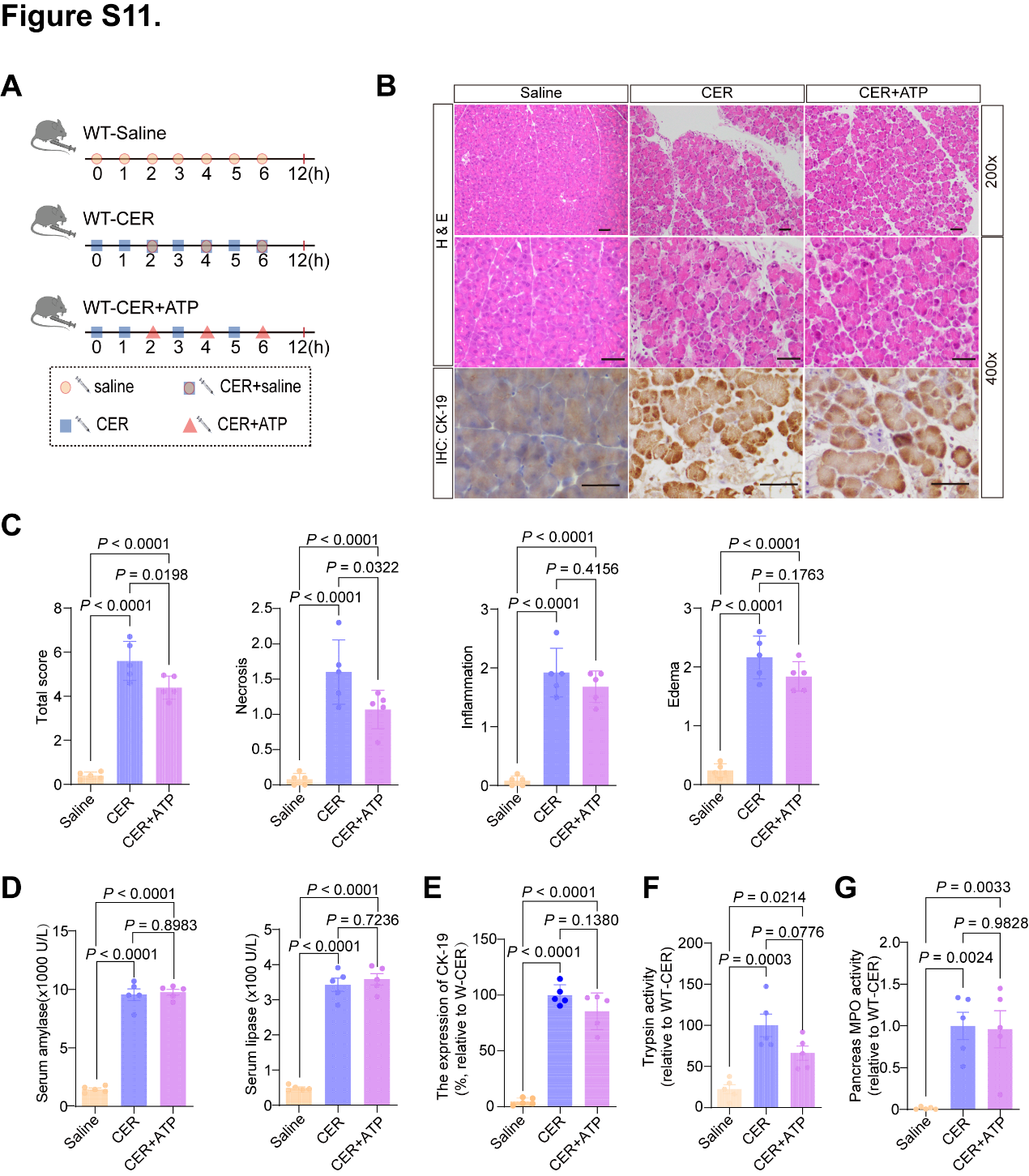


**Figure S11. ATP treatment was able significantly to reduce the cell necrosis in pancreatic tissues in wild-type mouse models.**

(A) The flow charts of experiment. (B) The representative histological and immunohistochemical images of the pancreas for CK-19 were obtained 12 hours after the first administration of CER (Scale bar, 50 μm). (C) The histological evaluation of pancreatic edema, inflammatory infiltration, necrosis, and the overall sum of these features. (D) The levels of serum amylase and lipase were measured at 12 hours after the first administration of CER. (E) Quantification of immunohistochemical analysis for CK-19 of mouse pancreatic tissues at 12 hours after the first administration of CER (F) Representative of trypsin activity in pancreatic tissues at 12 hours after the first administration of CER. (G) Representative of activity of MPO in pancreatic tissue at 12 hours after the first administration of CER. Data were from 5-10 mice per group and presented as mean ± SD from individual mice with one-way analysis of variance (ANOVA) and Tukey's multiple-comparison posttest.
